# Transcriptomic analysis in pediatric spinal ependymoma reveals distinct molecular signatures

**DOI:** 10.18632/oncotarget.23311

**Published:** 2017-12-14

**Authors:** Anbarasu Lourdusamy, Li Z. Luo, Lisa CD. Storer, Kenneth J. Cohen, Linda Resar, Richard G. Grundy

**Affiliations:** ^1^ Children’s Brain Tumour Research Centre, School of Medicine, Queen’s Medical Centre, University of Nottingham, Nottingham, UK; ^2^ The Johns Hopkins University School of Medicine, Baltimore, Maryland, USA

**Keywords:** pediatric ependymoma, gene expression, spinal ependymoma, microRNA

## Abstract

Pediatric spinal ependymomas (SEPN) are important albeit uncommon malignant central nervous system tumors with limited treatment options. Our current knowledge about the underlying biology of these tumors is limited due to their rarity. To begin to elucidate molecular mechanisms that give rise to pediatric SEPN, we compared the transcriptomic landscape of SEPNs to that of intracranial ependymomas using genome-wide mRNA and microRNA (miRNA) expression profiling in primary tumour samples. We found that pediatric SEPNs are characterized by increased expression of genes involved in developmental processes, oxidative phosphorylation, cellular respiration, electron transport chain, and cofactor metabolic process. Next, we compared pediatric spinal and intracranial ependymomas with the same tumours in adults and found a relatively low number of genes in pediatric tumours that were shared with adult tumours (12.5%). In contrast to adult SEPN, down-regulated genes in pediatric SEPN were not enriched for position on chromosome 22. At the miRNA level, we found ten miRNAs that were perturbed in pediatric SEPN and we identified regulatory relationships between these miRNAs and their putative targets mRNAs using the integrative miRNA-mRNA network and predicted miRNA target analysis. These miRNAs include the oncomiR *hsa-miR-10b* and its family member *hsa-miR-10a*, both of which are upregulated and target chromatin modification genes that are down regulated in pediatric SEPN. The tumor suppressor, *hsa-miR-124*, was down regulated in pediatric SEPN and it normally represses genes involved in cell-cell communication and metabolic processes. Together, our findings suggest that pediatric SEPN is characterized by a distinct transcriptional landscape from that of pediatric intracranial EPNs or adult tumors (both SEPNs and intracranial EPNs). Although confirmatory studies are needed, our study reveals novel molecular pathways that may drive tumorigenesis and could serve as biomarkers or rational therapeutic targets.

## INTRODUCTION

Ependymomas (EPNs) are primary tumours of the central nervous system (CNS) that affect both children and adults [1]. They account for 8-10% of CNS tumors in children and approximately 4% of adult CNS tumors [2, 3]. Current treatment strategies for malignant EPNs include maximal surgical resection, radiation therapy, and chemotherapy [4]. However, the benefits of chemotherapy following surgery remain unclear. Unfortunately, children with EPN have a relatively poor prognosis with a 5-year progression-free survival rate of only 14%, whereas the 5-year disease-free survival in adults with EPN approximates 70% [5]. EPNs are histologically classified into three major subtypes according to the World Health Organization (WHO), including: myxopapillary EPN or subependymoma (grade I), EPN (grade II), and anaplastic EPN (grade III) [6]. Current prognostic factors for EPNs are based on clinical and histological criteria, such as extent of tumor resection and histological grade [7]. Complete surgical resection is not always achieved and studies to investigate the prognostic value of the WHO grading system have yielded conflicting results. Therefore, there is an important and unmet need to delineate the cellular and molecular pathogenesis of EPNs in order to develop more robust prognostic signatures and to identify new therapeutic targets to improve outcomes.

EPNs develop throughout the entire CNS but occur predominantly in intracranial and spinal cord regions. Intracranial EPNs, especially from the posterior fossa (PF), are frequently found in children, whereas EPNs from the spine are more common in adults [8]. Histologically, EPNs within each of these anatomical locations have similar morphology, although recent genomic studies suggest that their molecular landscapes differ [9–11]. For example, PF EPNs represent at least three distinct subgroups based on DNA methylation profiles, including: 1) group A (PF-EPN-A), 2) group B (PF-EPN-B), and 3) subependymomas (PF-SE). Supratentorial (ST) EPNs can be further classified into subependymoma (ST-SE) and two subgroups characterized by fusion genes, including, *C11orf95-RELA* (ST-EPN-RELA) or *YAP1-MAMLD1* (ST-EPN-YAP1). Similarly, spinal ependymomas have been grouped into three molecular subgroups that appear to correlate with their histologic subtype, including: subependymomas (SP-SE), myxopapillary ependymoma (SP-MPE), and ependymoma (SP-EPN). The recent WHO 2016 classification has reflected these changes by incorporating the ST-EPN-RELA subgroups as a distinct entity [12]. Mutations in the *neurofibromatosis type 2* (*NF2*) gene or monozygosity of chromosome 22q were previously associated with SEPNs [13], but were limited to the SP-EPN subgroup and comprised predominantly of adult ependymomas. These genetic alterations are not exclusive to SEPNs as the loss of 22q was also observed in several other intracranial subgroups [10]. It should be emphasized that the number of pediatric SEPNs in the DNA methylation-based subgroup analysis is very low (n=2) compared to the adult SEPNs (n=53) [10]. Studies using gene expression profiling uncovered a number of genes and characteristic pathway alterations in SEPNs, including HOX genes family, CNS development and morphogenesis, angiogenesis, blood coagulation and glial cell differentiation [10, 11, 14]. However, the vast majority of these studies focused on spinal tumors from adult patients. Although prior research indicates that SEPNs are distinct from intracranial ependymomas [14, 15], there are no studies comparing SEPN in children. Importantly, existing epidemiologic data suggest that tumor location, genetic lesions, and outcomes differ between children and adults, thus underscoring the need for further study to elucidate the underlying biology and identify optimal treatment approaches for children with SEPN.

Unlike adults where ependymomas occur primarily in the spine, in children, SEPN constitutes less than 10% of all ependymomas. Moreover, SEPN in pediatric patients is more likely to be aggressive with higher rates of local recurrence and CNS metastasis, compared to adults [16]. Because these tumours are uncommon, the molecular basis of pediatric SEPN is poorly characterized. We hypothesized that elucidation of transcriptomics in pediatric ependymomas, specifically evaluating spinal and intracranial EPN, would be key to understanding the biology of these tumors.

## RESULTS

### Differential expression of genes defines pediatric SEPN

**Figure 1:**
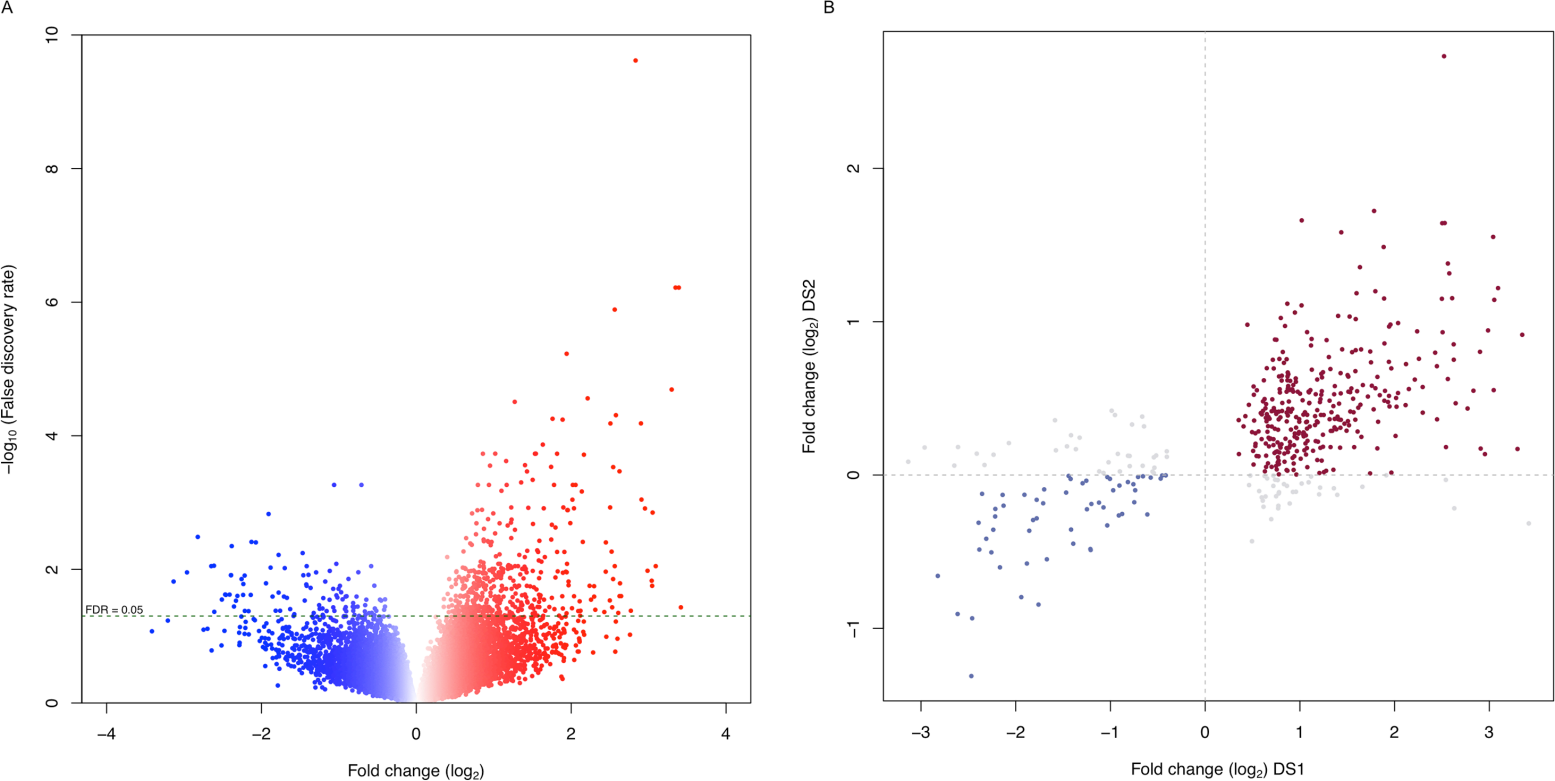
Gene expression profiles define distinct characteristics of pediatric SEPN **(A)** Volcano plot showing the number of significantly differentially expressed genes in pSEPN (FDR < 0.05). The x-axis represents expression fold change between pSEPN (n = 6) and pediatric intracranial ependymomas (n = 60) in log_2_ scale and the y-axis represents –log_10_ of false discovery rate (FDR). **(B)** Expression fold changes for all differentially expressed genes in this cohort are plotted on the x-axis against the fold changes (y-axis) for the same genes in the replicated dataset (DS2) with four SEPN and seventy-five intracranial ependymomas.

To identify molecular mechanisms underlying pediatric SEPN (pSEPN), we compared gene expression profiles of spinal (n = 6) and intracranial tumors (PF/ST, n = 30/30) from pediatric ependymoma patients (data set 1, DS1), selecting genes at a false discovery rate (FDR) < 0.05. We used an empirical Bayesian moderated t-test, which was previously shown to perform robustly on unbalanced microarray dataset. We identified a total of 557 differentially expressed genes between pediatric SEPN and intracranial ependymoma (Supplementary Table 1). Among them, 82% (455) of genes were over-expressed, while 102 were under-expressed in pSEPN (Figure 1A). This differential gene expression was consistent when we applied a nonparametric rank product method. The correlation between rank-product sum statistics and t-statistics from the empirical Bayesian method was high (|r| = 0.90 when pSEPN > intracranial EPN and |r| = 0.85 when pSEPN < intracranial EPN) and majority of the differentially expressed genes (90% of 455 over-expressed and 97% of 102 under-expressed) were within the top 15% of rank-product sum statistics (Supplementary Figure 1). We next investigated the effect of sample imbalance on differential expression. We randomly picked six samples each from PF/ST and performed differential expression analysis with samples from SP. We used an empirical Bayesian moderated t-test on equal number of samples and repeated this analysis with 100 different permutations of PF/ST samples. Remarkably, the direction of expression differences between SP and intracranial tumours was the same in all 100 permutations as in the initial observation with complete data for all 557 differentially expressed genes (Supplementary Figure 2A). In addition, for these 557 differentially expressed genes, the median Pearson correlation of t-statistics with the 100-permutated data was 0.913 (mean = 0.909, sd = 0.012, 25^th^ percentile = 0.906, 75^th^ percentile = 0.918) (Supplementary Figure 2B) indicating sample imbalance had no impact on the observed differences between SP and intracranial tumours. To test whether these findings were replicable, and to further validate these results, we examined differential expression in an independent data set (DS2). The DS2 consisted of expression data generated with Affymetrix Human Exon ST 1.0 microarrays from tumor samples of 79 pediatric ependymoma cases (SP = 4, PF/ST = 50/25). Though these arrays differ from the ones used in DS1, there was a positive correlation of test statistics for differential expression between DS1 and DS2 for the differentially expressed genes also present in the DS2 data (457 of 557; Pearson correlation *r* = 0.55, *P* < 2.2×10^−16^); the correlation remained significant (*r* = 0.27, *P* < 2.2×10^−16^) across all 14,097 genes common to both platforms after quality control (Supplementary Figure 3). Moreover, 84% of 457 genes in DS2 showed the same direction of expression differences between SP and intracranial tumours as in the initial cohort, DS1 (Figure 1B), supporting the robustness of the differential expression results. Next, we ascertained whether these differentially expressed genes are specific for pSEPN. We therefore obtained expression data from six publicly available studies (Supplementary Table 2), selected those tumor samples of patients with age < 18 years from ST or PF EPNs (n = 223 primary tumours), and analysed using a random-effect model to integrate gene-specific expression changes between ST and PF in each individual study. We identified 628 genes that show high expression in pediatric ST tumours and 364 genes with high expression levels in PF tumours (Unpublished data). Remarkably, there was no significant overlap observed between differential expressed genes in pSEPN and pediatric ST tumours (12/628 genes overlapped, P = 0.97, Fisher’s exact test) or between pSEPN and pediatric PF tumours (14/364 genes overlapped, P = 0.18, Fisher’s exact test). Thus, differential expression analysis produced robust and reproducible results, warranting further analysis.

### Gene ontology highlights genes linked to developmental processes and mitochondrial metabolism

**Figure 2:**
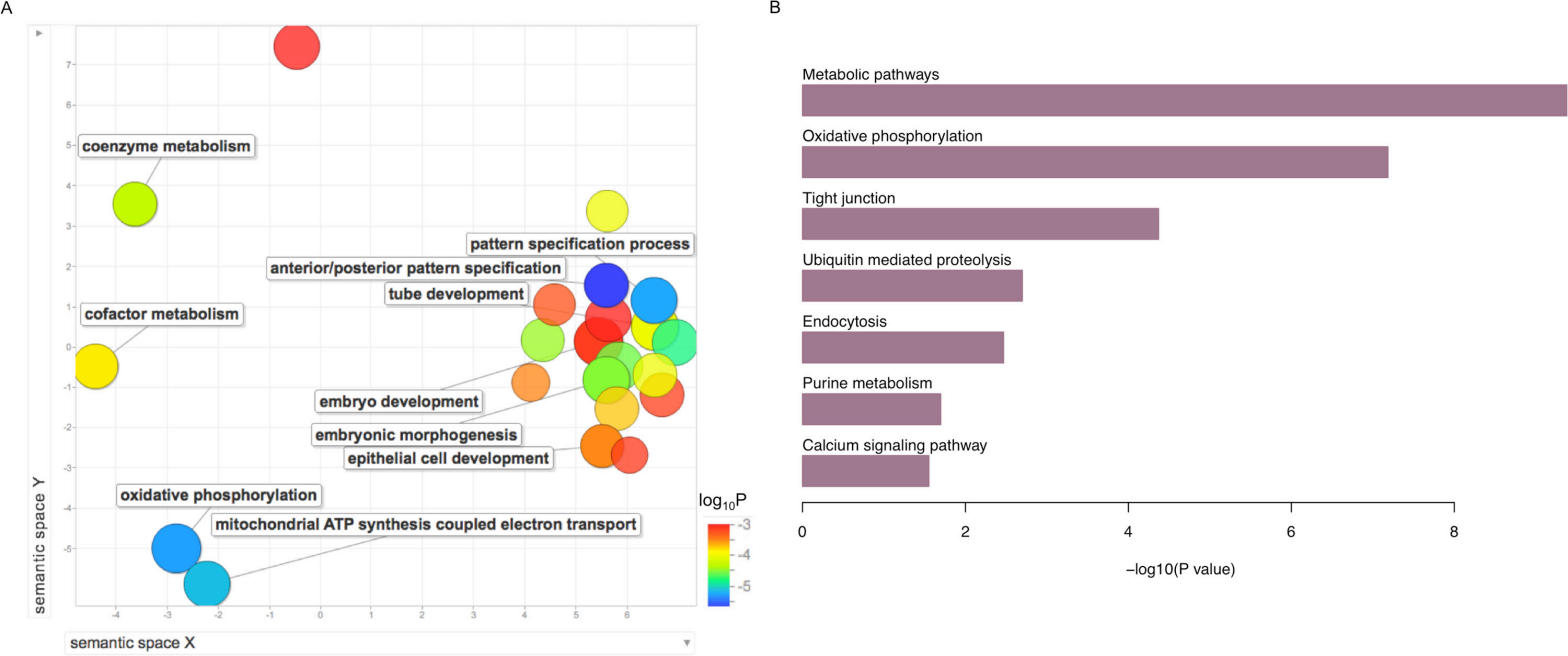
Summary of gene ontology (GO) biological processes and KEGG pathways derived from the enrichment analysis of upregulated genes in pSEPN **(A)** X- and Y-axes represent a two-dimensional annotation space derived from a multi-dimensional scaling procedure used on a matrix of GO terms’ semantic similarities. By employing this visualization method, similar biological categories will cluster together. Bubble color represents the p-value obtained from GO enrichment analysis and bubble size relates to the frequency of GO terms in the GO annotation database. **(B)** Results of enrichment analyses KEGG pathways for the upregulated genes in pSEPN is represented in the bar plots. We used the Hypergeometric test to calculate P values and used the FDR of 5% to obtain significantly enriched pathways.

To obtain a functional overview, we annotated the upregulated genes in pSEPN separately, using WebGestalt 2013 tool [17]. These genes are significantly enriched for a spectrum of gene ontology (GO) terms in biological processes category (Figure 2A and Supplementary Table 3). Pediatric SEPN demonstrated increased expression of genes involved in anterior/posterior pattern specification (17 genes; FDR = 1.0×10^−03^), embryonic morphogenesis (26 genes; FDR = 1.5×10^−03^), tube development (23 genes; FDR = 2.5×10^−03^), and epithelial cell development (8 genes; FDR = 8.7×10^−03^). In addition to development-related processes, we uncovered novel pathways that had not been linked to pSEPN. For example, we found up-regulation of genes involved in oxidative phosphorylation (9 genes; FDR = 1.5×10^−03^), cellular respiration (13 genes; FDR = 1.9×10^−03^), electron transport chain (12 genes; FDR = 3.1×10^−03^), and cofactor metabolic process (16 genes; FDR = 3.5×10^−03^). The mitochondrial oxidative phosphorylation (OXPHOS) system consists of five protein complexes and genes encoding components of complex I (*NDUFAB1, NDUFA8, NDUFB3, NDUFB5* and *NDUFB10*), complex II (*SDHD*), complex V (*ATP5H, ATP5J, ATP5O, ATP5S, ATP6V1G1* and *PPA2*), and coenzymes (*COQ7* and *COQ9*) showed increased expression in pSEPN. In addition, we found that mitochondrial biogenesis genes, in particular, the mitochondrial ribosomal genes *MRPL15, MRPL48, MRPL50, MRPS18C, MRPS33* and *MRPS6*, were also expressed at significantly higher levels in pSEPN when compared with pediatric intracranial ependymomas. Collectively, these results implicate mitochondrial metabolism and OXPHOS in the pathogenesis of pSEPN.

Next, we probed biological and disease pathways using the Kyoto Encyclopedia of Genes and Genomes (KEGG) database and found that metabolic, tight junction, ubiquitin mediated proteolysis, endocytosis, purine metabolism, and calcium-signalling pathways were enriched in genes differentially expressed pSEPN (Figure 2B). Together, these significantly over-represented pathways suggest that pSEPNs develop in the setting of complex biologic processes and point to perturbations in mitochondrial function and cellular metabolism as key underlying features.

### Comparative gene expression analysis of pediatric and adult SEPN

**Figure 3:**
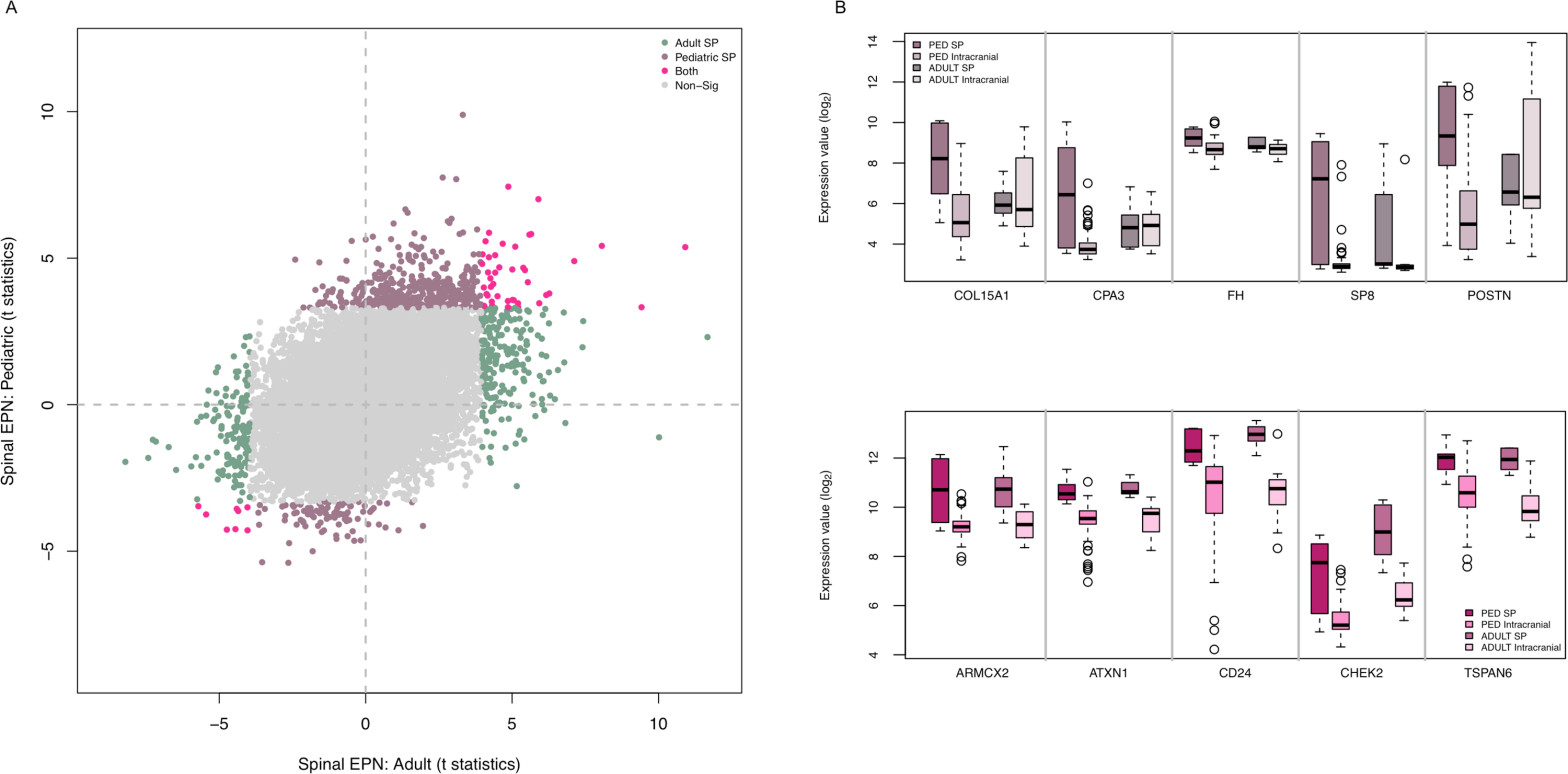
Pediatric SEPNs show distinct expression characteristics from adult SEPNs **(A)** Adult (x-axis) and pediatric (y-axis) moderated t-statistics of differential expression for each gene (point) are plotted when spinal EPNs were compared with intracranial ones. The number of these genes differentially expressed with both adult and pediatric (pink), in adult EPNs (green) and only in pediatric EPN (light brown). **(B)** Box plots showing log_2_ expression levels (y-axis) of representative (five) genes that show significant high expression only in pSEPN (top) and significant high expression in both pediatric and adult SEPN (bottom) when compared to pediatric intracranial ependymomas as well as to adult SEPN. PED SP (n = 6): pediatric spinal, PED intracranial (n = 60): pediatric intracranial, ADULT SP (n = 6): adult spinal, and ADULT intracranial (n = 11) ependymomas.

**Figure 4:**
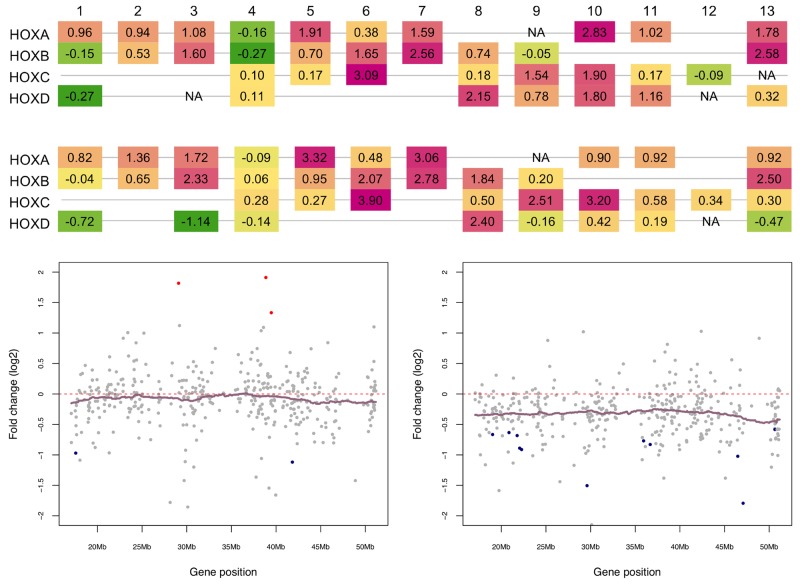
Distinct expression patterns of HOX genes and Chromosome 22 genes in pediatric and adult SEPN **(A)** Aberrant expression HOX genes in pediatric and adult SEPN. Schematic diagram of expression changes (fold change on log_2_) of HOX genes in pediatric (top) and adult (bottom) SEPN is displayed (green indicates under-expression whereas pink is for the over-expression in SEPN compared to intracranial ependymomas). HOX genes are arranged by paralogous group (1-13) in four clusters (A-D), each at a different chromosomal location. The expression of 15 HOX genes was significantly altered in pediatric whereas seven were found in adult SEPN (FDR < 0.05). In pediatric SEPN: *HOXA7, 10, 11*, and *13*; *HOXB6, 7, 8*, and *13*; *HOXC6, 9*, and *10*; *HOXD8, 9, 10*, and *11* were overexpressed. In adult SEPN: *HOXA7*; *HOXB6, 7*, and *8*; *HOXC6, 9*, and *10* were overexpressed. **(B)** Expression levels (log_2_) of chromosome 22 genes that are detected in our study for pediatric (left panel) and adult (right panel) SEPNs. Genes are sorted according to their position on the chromosome (x-axis) and colored according to the statistical significance of their expression level (y-axis). Significantly downregulated genes in SEPN (FDR < 0.05) are marked with blue, upregulated ones with red, and non-significant ones with grey. Normalized log_2_ expression values (dots) and kernel-smoothed expression values (purple line) are shown.

To begin to determine whether transcriptional networks in pSEPN overlap with those in adult tumours, we compared the expression profiles of pediatric to adult SEPN (n = 6 for SP and n = 4/7 for PF/ST). Remarkably, 87.5% of 400 differentially expressed genes were specific to adult SEPN, while only 12.5% were shared between adult and pediatric SEPN, suggesting distinct gene regulatory programs define these two tumour groups (Figure 3A and Supplementary Table 4). For example, *CPA3* showed high expression with pediatric SEPN (log_2_ fold change (FC): 2.54; FDR = 2.94×10^−04^) but not with adult SEPN (FC: 0.001; FDR = 0.99) when compared with intracranial tumours (Figure 3B). The shared list of up-regulated genes in adult and pediatric SEPN included genes involved in DNA damage and signal transduction resulting in induction of apoptosis (*CHEK2*), negative regulation of IGF1 receptor signalling pathway (*ATXN1* and *CLIP*), and extracellular matrix constituent secretion (*CTGF*) (Figure 3B and Supplementary Table 4). In addition, seven protein coding HOX genes and a non-protein coding HOXB cluster antisense RNA 3 (*HOXB-AS3*) showed significant changes in expression in both pediatric and adult SEPN (Supplementary Table 4). HOX genes from groups 5 to 13 show elevated expression in cervical and lumbar regions of the spinal cord [18], and higher expression levels of seven genes from this group in adult SEPN further validate the importance of HOX genes in EPN. Remarkably, no other HOX genes were found among the remaining 203 up-regulated genes specific to adult SEPN. Several differentially expressed *HOX* genes were significantly up regulated in pediatric, but not in adult SP EPN, including *HOXA* (*A10, A11* and *A13*), *HOXB (B13)*, and *HOXD (D8, D9, D10* and *D11*) (Figure 4A). Interestingly, we also identified several genes whose expression was up-regulated in pediatric but down-regulated in adult SEPN, including genes involved in developmental processes (*ARX, CNTF, FOXE1, HOXD9* and *LHX9*), genes associated with lipid (*AMT, CERS3, HPGD* and *OXNAD1*), carbohydrate (*GTPBPB* and *PTGR1*) and RNA metabolic processes (*CSTF3, TLE4* and *ZNF382*), and regulation of cell proliferation (*AGTR1*).

Loss of chromosome 22 and monozygosity for 22q with frequent mutations in a tumour suppressor gene, *neurofibromin 2* (*NF2*) have been detected in the SEPN genome [10, 13]. In the current study, the *NF2* gene was downregulated in both pediatric and adult SP EPN compared to intracranial ependymomas; however, the change in expression was not significant after correction for multiple testing (Supplementary Figure 4). To examine whether loss of regions on one or both chromosomes could repress expression of genes located on chromosome 22 in pSEPN, we performed the positional gene enrichment (PGE) analysis to identify chromosome regions significantly enriched in differentially expressed genes [19]. There were 91 significantly downregulated genes in pSEPN and PGE analysis showed enrichment of genomic regions on several chromosomes, but not on chromosome 22 (Supplementary Table 5). By contrast, down-regulated genes in adult SEPN show significant enrichment for several chromosomes, including genomic regions on chromosome 22 (20101693-20637217, FDR = 2.24×10^−04^; 20381828 – 20637217, FDR = 8.79×10^−04^; 34267296 – 35113958, FDR = 4.95×10^−03^; and 44277383-45512816, FDR = 4.95×10^−03^). As a proof of the robustness of our findings, we plotted the change in expression (fold-change on log_2_ scale) between SEPN and intracranial ependymomas in both pediatric and adult for all chromosome 22 genes, and found similar qualitative findings (Figure 4B). We observed that chromosome 22 has the highest percentage of down-regulated genes in adult SEPN (∼84%) compared to pSEPN (∼58%) and, in particular, the enriched q11 band contains genes *DGCR2, HIC2, MED15, PPM1F* and *YPEL1* that were significantly down regulated in adult SEPN (FDR < 0.05). These data suggest that dosage imbalance of chromosome 22 genes occurs in adult SEPN, but not in pSEPN. Together, these findings illuminate distinct gene expression patterns of pSEPN versus adult SEPN and provide evidence for potentially pathogenic changes in gene expression.

### Differential expression of miRNAs in pediatric SEPN

To obtain a comprehensive list of candidate miRNAs that are characteristic of pSEPN, we compared the expression of each miRNA in SEPN samples (n =6) with those of intracranial tumours (n = 43) obtained from pediatric ependymoma patients. Global expression was measured with the Agilent human miRNA microarray that contains probes for 723 human microRNAs and 76 human viral microRNAs from the Sanger database v.10.1. After normalizing microarray data, we excluded all nonhuman miRNAs, and miRNAs not detected in 10% of total samples, leaving 311 miRNAs detected. To find differentially expressed miRNAs between SP and intracranial sample groups, two-sample moderated *t* statistic was performed on each miRNA. There were six up- and four down-regulated miRNAs in SP relative to intracranial tumors (FDR < 0.05) (Table 1). Several differentially expressed miRNAs in pSEPN were already known to function as “oncomiRs” (*has-miR-10b* and *has-miR-196a*) or “tumour suppressors” (*hsa-miR-26a* and *hsa-miR-124*), suggesting their potential role in the pathogenesis of these tumors. *miR-10b* was the most significantly induced, displaying >10-fold increase in pSEPN. Another miR-10 family member, *miR-10a*, was also enriched (∼ 4-fold). As *miR-10a* resides in the *HOXB*-gene cluster on chromosome 17 and *miR-10b* resides in the *HOXD*-gene cluster on chromosome 2, it is likely that miR-10 family members play important roles in regulating multiple processes during brain development, and alterations in their functions may lead to initiation or progression of pSEPN.

**Table 1:** The dysregulated microRNAs in pediatric SEPN (n = 6) compared to pediatric intracranial ependymomas (n = 43)

miRNA ID	FC (log_2_)	Mean expression (log_2_)	FDR
**Up regulated**			
hsa-miR-10b-5p	3.40	5.08	1.36×10^−02^
hsa-miR-196a-5p	2.55	2.66	1.36×10^−02^
hsa-miR-10a-5p	1.94	5.43	2.36×10^−02^
hsa-miR-144-5p	1.74	4.20	2.36×10^−02^
hsa-miR-23b-3p	1.22	10.38	2.36×10^−02^
hsa-miR-27b-3p	1.13	10.63	2.36×10^−02^
**Down regulated**			
hsa-miR-124-3p	−3.34	6.19	4.55×10^−02^
hsa-miR-153-3p	−2.40	5.61	4.56×10^−02^
hsa-miR-885-5p	−2.18	5.98	2.36×10^−02^
hsa-let-7b-5p	−1.05	12.49	5.33×10^−02^
hsa-miR-26a-5p	−0.66	11.49	2.36×10^−02^

FC (log_2_): Fold change in expression between pediatric SP EPN and intracranial ependymomas on log_2_ scale; Mean expression (log_2_) across all tumor samples; and FDR: false discovery rate.

### Relationship between miRNA and mRNA expression changes

Protein-coding transcript levels can be post-transcriptionally regulated by the activity of miRNA. These small RNA molecules silence mRNA translation through sequence-specific targeting. To understand miRNA-guided regulation, it is essential to uncover the downstream mRNA targets in addition to profiling miRNA identities. This prompted us to analyse miRNA-mRNA relationships in the context of pSEPN. We focused this miRNA-mRNA analysis on the 10 differentially expressed miRNAs that we identified above. We first used TargetScan [20], miRanda [21], and miRDB [22] databases to find putative miRNA targets based on sequence complementarity to mRNA 3′ UTR of reliably expressed protein-coding genes. We next compared the expression profiles between miRNA-mRNA pairs and selected the anti-correlated pairs with statistically significant association (FDR < 0.05). The combination of the correlation and target prediction filters yielded 159 miRNA-mRNA pairs (∼ 6.6% of miRNA-mRNA pairs detected from the expression data at FDR < 0.05), of which 56 pairs were related to miRNAs that were downregulated in pSEPN (Supplementary Table 6). A GO enrichment analysis on the set of 159 high-confidence miRNA targets indicated enrichment in broad categories, including developmental processes, multicellular organism signalling, cytoskeletal protein binding and transport functions, consistent with aberrant development in pSEPN and the regulatory role of the miRNA machinery in orchestrating these functions (Supplementary Figure 5). Remarkably, the tumor suppressor miRNA, *hsa-miR-124*, targeted the majority of detected mRNA targets (75% of 56) that were involved with developmental processes (*EFNB3, NR4A3, PAX3* and *SYNOP2*), cell-cell communication (*AFF1, RALGPS2* and *TSPAN6*), and metabolic processes (*CUL5, GRSF1, SGMS2, STK35* and *ZFAND3*). The *hsa-miR-153*, which was the second most down-regulated miRNA in pSEPN, targets genes encoding transporter proteins (*ITPR1, MATN2* and *SLC44A1*), calcium-binding protein (*CIB2*), transcription factor (*CITED2*), and a receptor, *TGFBR2*, which showed significant up-regulation in pSEPN (log_2_ Fold change, FC = 1.50, FDR = 0.034). Interestingly, *Matrilins* 2 (*MATN2*) was co-ordinately targeted by both *hsa-miR-124* and *hsa-miR-153*.

**Figure 5:**
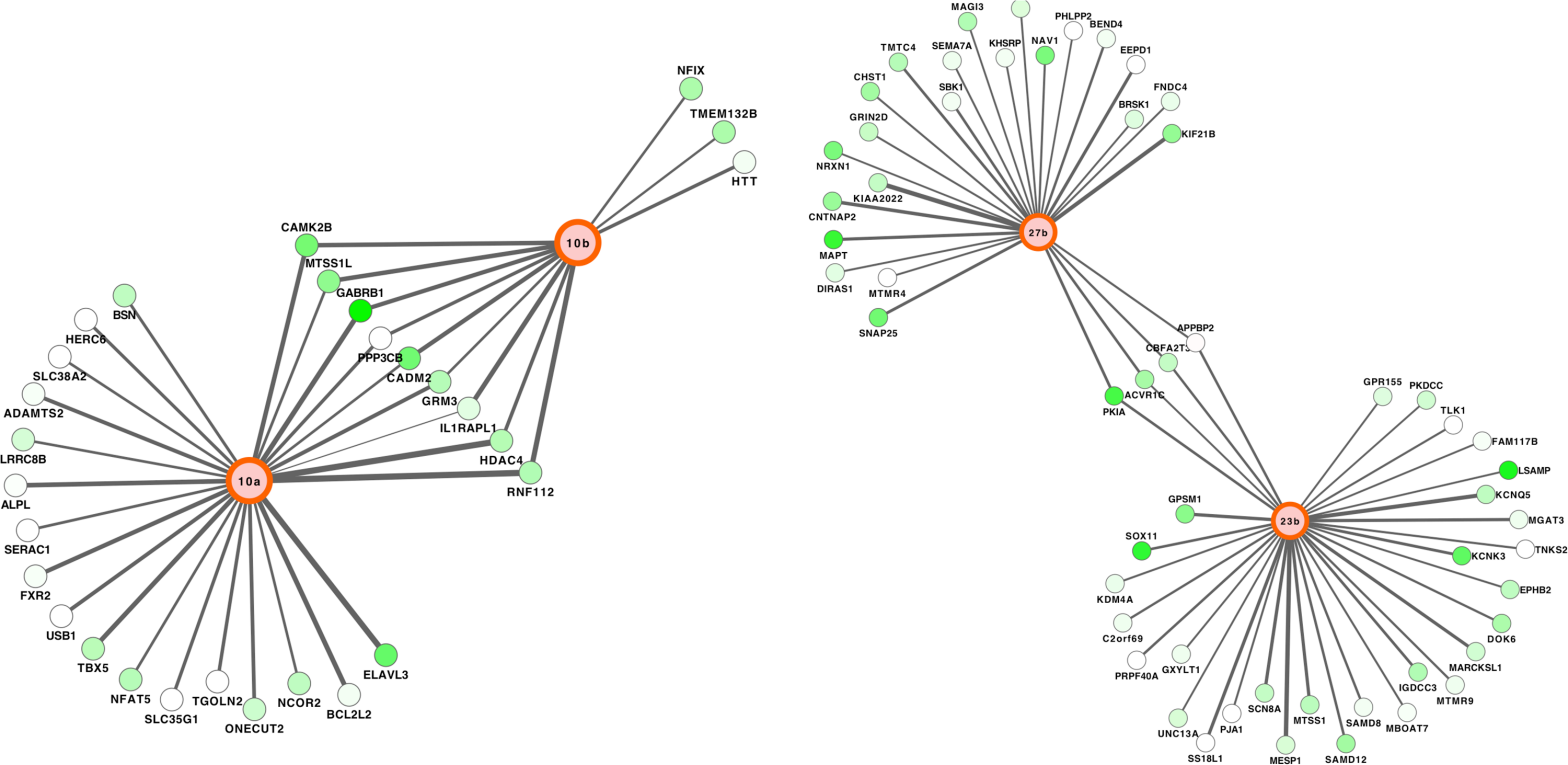
Networks of miRNA-mRNA target interactions in pediatric SEPN Two highly connected sub-networks from the inferred network of pediatric EPNs comprising 159 putative target interactions between miRNAs and mRNAs. Edge width represents strength of Spearman rank correlation for a given miRNA-mRNA pair, green colour represents evidence of significant downregulation in pSEPN.

We next focused our attention on the putative targets of the most abundantly expressed miRNAs in pSEPN to increase our confidence in any possible miRNA-mRNA regulatory interactions. The putative mRNA targets of up-regulated miRNAs were highly enriched for synaptic transmission (*CNTNAP2, DIRAS1, GPSM1, GRM3, HTT* and *SNAP25*), chromatin modification (*HDAC4, KDM4A, SS18L1* and *TLK1*), and negative regulation of transcription from RNA polymerase II promoter (*HDAC4, NCOR2, NFIX, PKIA* and *SOX11*). Interestingly, genes involved with cell differentiation (*ACVR1C, CBFA2T3, ELAVL3, GPSM1* and *PKDCC*) were also repressed by upregulated miRNAs in pSEPN. The top most upregulated miRNA in pSEPN, *hsa-miR-10b*, and its closely related family member, *hsa-miR-10a*, cooperatively target several genes including those encoding synaptic cell adhesion molecule (*CADM2*) and histone deacetylase 4 (*HDAC4*) that play a critical role in transcriptional regulation, cell cycle progression, and developmental events (Figure 5). The transcriptional co-repressor gene, *CBFA2T3*, stimulates mitochondrial respiration. The gene encoding Activin A receptor type 1C (*ACVR1C*), acts as an anti-proliferative factor during early stages of oncogenesis, and is preferentially targeted by *hsa-miR-23b* and *hsa-miR-27b*, which are located in one genomic cluster (miR-23b/27b). The G-Protein Signalling Modulator 1 (*GPSM1*) and the protein kinase, *PKDCC* were targeted by *hsa-miR-23b*, while the neural-specific RNA-binding protein, *ELAVL3* was targeted by *hsa-miR-10a* (Figure 5).

## DISCUSSION

Our comprehensive analysis on the pSEPN transcriptomics landscape provides novel insights into putative molecular mechanisms underlying pSEPN. Based on gene expression signatures, including both protein-coding genes and miRNAs, we discovered significant up-regulation in specific genes and their associated pathways in pSEPN when compared to pediatric intracranial ependymoma or adult EPN. Moreover, the differential expression of a subset of genes expressed in pSEPN compared to pediatric intracranial EPN was validated in an independent cohort of pediatric EPNs. Together, these findings suggest that this gene signature is therefore likely to be contributes to the underlying biology of these tumours.

Surprisingly, among genes with increased expression in pSEPN, there was strong enrichment for mitochondrial genes (63 genes; P = 2.58×10^−08^) related to oxidative phosphorylation, electron transport chain and cofactor metabolic process, all of which had not been previously linked to pSEPN. The oxidative phosphorylation (OXPHOS) system is composed of NADH:ubiquinone oxidoreductase (complex I), succinate dehydrogenase (SDH; complex II), coenzyme Q cytochrome *c* oxidase (complex III), cytochrome *c* oxidase (COX; complex IV), ATP synthase (complex V), and 2 electron carriers, namely cytochrome *c* and coenzyme Q [23]. Genes encoding protein complexes I, II and V as well as coenzymes and proteins involved in mitochondrial biogenesis showed elevated expression in pSEPN. In addition, contrary to the results of recently published study [24], none of the genes characteristic of the ‘Warburg phenotype’ (*HIF1A*: FC = −0.02, FDR = 0.970; *HK1*: FC = −0.15, FDR = 0.727; *HK2*: FC = −1.07, FDR = 0.197; and *PDK1*: FC = −0.39, FDR = 0.745) showed increased expression in pSEPN when compared to pediatric intracranial EPN. It should be emphasized that the prior study on ‘Warburg phenotype’ assessed 35 SEPNs (∼80% from adult ependymoma patients), comparing gene expression in the myxopapillary ependymoma (MPE) subtype to that of Grade II SEPNs, and found that genes related to ‘Warburg phenotype’ were up-regulated in MPE [24]. Together, our findings demonstrate enhanced expression of genes involved in oxidative energy metabolism with a shift away from glycolytic genes towards those involved in OXPHOS in pSEPN compared to pediatric intracranial EPN. Investigations aimed at understanding the mechanisms of this program deserve further attention.

As expected, we also found strong enrichment of developmental genes in pSEPN, including those associated with anterior/posterior pattern specification, embryonic morphogenesis, tube development, and epithelial cell development. Notably, HOX genes that play fundamental roles in specifying the anterior-posterior (A-P) body patterning and spinal cord development were significantly upregulated in pSEPN. The 39 human HOX genes are divided into four groups (A–D), which are further divided into paralogue groups (1–13) based on their position numbered from the 3′ to the 5′ end (1 to 13), with the 3′ end genes expressed the earliest and linked to the development of rostral structures whereas the 5′ end genes linked to the development of more caudal structures [18]. It is interesting to note that all HOX genes (6 to 13) that showed high expression in pSEPN correspond to HOX genes that are associated with caudal structures (Figure 3). It is also of interest that the expression patterns of some of these HOX genes located closest to the 5′ end of the cluster are preserved in pediatric and adult SEPN, indicating spatial relationships of HOX-expressing spinal cord remain unchanged during the course of development. This characteristic expression pattern of *HOX* genes in adult and pediatric SEPNs further support their important role in regional identity and the maintenance of spinal cord specificity. Genes in the HOX family have been the focus of investigation in a variety of malignancies, as they play important roles in stem cell renewal, cellular fate determination, and body-pattern development [25, 26]. Specifically, HOX cluster 10 −13 is associated with normal development of the lumbosacral region. Moreover, prior studies revealed oncogenic function of *HOXA11, HOXA13*, and *HOXD11* in other cancers, implicating these genes in the pathogenesis of SEPN, although further studies will be needed to elucidate their role in this setting.

Emerging evidence suggests that pediatric and adult central nervous system tumors are biologically distinct entities [27], and our results provide additional support for this concept. The relatively low numbers of differentially expressed genes that were shared between adult and pediatric SEPN indicate distinct transcriptional programs. In comparison to adult ependymomas, pSEPN tumors show a remarkable convergence on genes associated with developmental processes and mitochondrial metabolism. Indeed, subsets of genes that are typically repressed in adult EPN are overexpressed in pSEPN. In contrast, deletions in chromosome 22, including *NF2* mutations, are among the most commonly reported genetic abnormalities in SEPN [10, 13]. Our analysis suggested that loss of chromosome 22 occurs in adult rather than in pSEPN, which likely has important functional consequences in the pathogenesis of adult SEPN. The differences between pediatric and adult SEPN may be partly related to the limitations inherent to the rather small number of cases in both comparisons, particularly the lower incidence of intracranial EPN in adults. There were also a smaller number of SEPN in pediatric cases. These issues need to be addressed in the future by studying a large series of cases using uniform criteria.

Because pediatric CNS tumors have fewer mutational events relative to their adult counterparts, it is critical to investigate factors that regulate the transcriptome, such as miRNAs, in order to gain a comprehensive understanding of tumorigenesis. Differential expression analysis uncovered six miRNAs whose expression levels are elevated in pSEPN. Of these, three (miR-10a, miR-144^*^ and *miR-196a*) reside on chromosome 7 and two on chromosome 9 (*miR-23b* and *miR-27b*). Precursors of *miR-10a* and *miR-196a* (∼50 kb apart) and *miR-23b* and *miR-27b* (0.141 kb apart) are closely clustered in the genome, suggesting common upstream transcriptional controls. As *miR-10a* shares the same seed sequence with *miR-10b*, the most abundant and differentially expressed miRNA in pSEPN, and reside within the HOX gene cluster (10a in *HOXB* whereas 10b in *HOXD*), it is likely that miR-10 family members play key roles during development. Dysregulation of miR-10 family members has been reported for several human cancers, including up-regulation of both *miR-10a* and *miR-10b* in glioblastoma and anaplastic astrocytomas, in some cases reaching more than a 100-fold overexpression, suggesting an oncogenic potential in brain tumors [28, 29].

Integration of miRNAs and mRNA expression profiles with the targeting information obtained from databases allowed us to identify a set of regulatory miRNA-mRNA pathways that may play a role in the pathogenesis of pSEPN. For the 159 high-confidence miRNA-mRNA target pairs, assignment of GO terms indicated that the pSEPN miRNAs extensively regulate developmental processes, especially cell differentiation, nervous system development, and anatomical structure development. Among distinct molecular functions, the pSEPN miRNAs target a large number of transcripts responsible for cytoskeletal protein binding, kinase activity, and ion transporter activity. Genes located in membrane and synapse, as well as neuron projection that are crucial in regulating the transmission of information through the spinal dorsal horn occupy the largest proportion of miRNA targets in the “cellular component” category. Interactions of individual miRNAs are summarized in Supplementary Table 5, which provides clues to how a particular miRNA may contribute to regulating transcripts, which in turn, distinguish pSEPN from pediatric intracranial EPN. Several individual miRNA-target relationships in the high-confidence set illustrate the potential relevance to the developmental and metabolic processes in the pSEPN. miR-124, which is one of the most strongly and uniformly down-regulated miRNAs in brain neoplasia, targets the *GRSF1* transcript (Supplementary Table 5). The protein encoded by this gene belongs to a family of ubiquitously expressed RNA-binding proteins that play key roles in all steps of posttranscriptional regulation of RNAs. Loss of *GRSF1* results in a specific protein synthesis defect with failure to assemble the required levels of oxidative phosphorylation complexes, implicating *GRSF1* as a key regulator of posttranscriptional mitochondrial gene expression [30]. Additionally, *hsa-miR-124-3p* was shown to target the *tetraspanin 6 (TSPAN6)* gene, which encodes a cell surface glycoprotein protein that mediates several signal transduction events. *TSPAN6* interacts with nuclear receptor subfamily 4 group A member 3 (*NR4A3*) protein, which modulates numerous processes, including CNS development, cell differentiation and lipid metabolism. The protein encoded by the *MATN2* gene is involved in axonal guidance and was significantly elevated in pSEPN (log_2_ fold change, FC = 2.43, FDR = 0.043). *MATN2* is a putative target of both *hsa-miR-124-3p* and *hsa-miR-153-3p*. Of note, the MATN2 protein is elevated in NF1-associated pilocytic astrocytoma with an unusually aggressive clinical phenotype [31]; it will therefore be interesting to further examine the effects of *MATN2* and potential regulation by *miR-124* and *miR-153* in SEPN. Future efforts are warranted to elucidate the functional roles of the novel transcriptional networks revealed by our studies.

When studying a rare cancer such as pSEPN, adequate sample size is an inherent challenge, and was a limitation of this study. Nonetheless, our study revealed that pSEPN is associated with increased expression of genes involved in oxidative phosphorylation. As expected, we also found overexpression of diverse developmental pathway genes. In addition, there was a striking up-regulation in expression of the oncogenic miRNA, *miR-10b*, and its closely related family member, *miR-10a* in pSEPN. This integrated miRNA-mRNA analysis reveals a comprehensive genomic landscape of pSEPN and provides the groundwork necessary to design larger, multi-centre studies to validate the cancer transcriptome as well as uncover novel biomarkers and therapeutic targets for pSEPN.

## MATERIALS AND METHODS

### Microarray data acquisition and analysis

Both mRNA and miRNA expression profiling of pediatric ependymoma were part of our published study [9]. The raw data was obtained from the Gene Expression Omnibus (GEO) database (GSE21687). This study contains tumor samples from 66 pediatric EPN patients (SP: 6 and PF/ST: 30/30) with a mean age of 6.47 (+ 4.60) years. The mRNA data were generated using the Affymetrix Human Genome U133 Plus 2.0 arrays. Expression intensity values were calculated at probeset level for the 66 CEL files using the robust multi-array average (RMA) method [32]. Probesets that are ‘absent’ (present/absent call using MAS5) in all samples were filtered out from the analysis. Expression values were mapped from probeset to unique gene and the probeset with the highest mean expression value was selected when multiple probesets were mapped to the same gene. The final filtering step left a total of 18,365 genes. For the validation of differentially expressed genes, we used an additional dataset (GSE27279) of mRNA microarrays from 79 pediatric EPN patients (SP = 4, PF/ST = 50/25) with a mean age of 5.93 (+ 4.70) years [11]. The data were generated using the Affymetrix human Exon 1.0 ST arrays. We used the Affymetrix Power Tools to generate gene-level (core meta-probeset) expression values from raw CEL files. Arrays were normalized using RMA, which included RMA background correction, quantile normalization, log transformation, and probeset summarization. Detection above background (DABG) was performed at both the probe and the probeset level using GC-matched background probes, and low variance probesets were excluded (17,001 genes). To identify differentially expressed genes, linear models were fitted with Bioconductor’s limma package [33], which uses a moderated t-statistic based on empirical Bayesian method and the P values were adjusted using Benjamini and Hochberg FDR procedure [34]. In addition, we used Rank Product, a nonparametric method designed for experiments with a small number of replicates [35]. Differential expression was defined for genes with FDR < 0.05.

miRNA expression was profiled by Agilent microarrays, and processed and normalized using the AgiMicroRna R package (using between-array quantile normalization) [36]. To filter miRNAs with very low expression across most EPN samples, we removed miRNAs that were detected in < 25% of samples (using the ‘detected’ flag in the microarray data sets). The final expression datasets contain 300 miRNAs. We used a moderated t-statistic based on empirical Bayesian method to identify differentially expressed genes or miRNAs between pediatric spinal and other ependymomas [33]. The p-values obtained from moderated t-test were corrected for multiple hypotheses using Benjamini and Hochberg algorithm [34]. The corrected P value, False Discovery Rate (FDR) less than 5% was used to select the differentially expressed genes or miRNAs.

### Gene set enrichment and pathway analysis

The over-representation analyses for Gene Ontology (GO) terms, Kyoto Encyclopedia of Genes and Genomes (KEGG), and Panther pathways, were carried out with WebGestalt 2013 tool [17]. The REVIGO software was used to summarize and visualize significant GO terms in a two-dimensional space as a scatter plot, derived by applying multidimensional scaling to a matrix of the GO terms’ semantic similarities using the SimRel algorithm [37]. In the scatter plot, bubble colour indicates the p-value obtained from the WebGestalt’s over-representation analysis and size indicates the frequency of the GO term in the underlying GO database (bubbles of more general terms are larger). The overlap between differentially expressed genes and chromosomal positions was investigated using the positional gene enrichment analysis tool [19]. The significance of statistically enriched functional categories, pathways, and gene sets was estimated either with hypergeometric test or with Fisher’s exact test and the p-values were corrected for the multiple comparisons by estimating the FDR.

### miRNA network analysis

Human miRNA target predictions were obtained from three different databases: miRanda-miRSVR (August 2010 release, http://microrna.org), TargetScan (Release 7.0, August 2015, http://targetscan.org) and mirDB (Version 5, Release date Auguest 2014, http://mirdb.org) [20–22]. We used miRanda-miRSVR scores, evolutionary conservation scores, and TargetScan context scores aggregated per gene and miRNA. Predicted miRNA targets are defined by the intersection of miRanda (score < −0.5 and conservation score > 0.5), TargetScan (context-score < −0.2) and miRDB (Target score > 50), unless otherwise stated. We used these thresholds to obtain a high-confidence list of predicted miRNA-target interactions. To identify the functional regulations from miRNAs to mRNAs, we combined both the computational target predictions at the sequence level and the inverse expression relationships between miRNAs and mRNAs in the context of EPN. The Spearman rank correlation coefficients were calculated for each miRNA–mRNA pair, p-values were corrected for multiple hypotheses, and significant correlations were selected at *FDR* < 0.05. Finally, the functional regulations were detected using miRNAs and genes characterized by (i) the strongest targets, which have the highest predicted targeting efficacy (ii) the most conserved target sites, which are more likely to have conserved physiological roles, but may not include newly evolved targets with species-specific functions, and (iii) strong correlation from miRNA-mRNA expression profiles.

## SUPPLEMENTARY MATERIALS FIGURES AND TABLES




