# Supplementary material for: Transcriptomic analysis in pediatric spinal ependymoma reveals distinct molecular signatures
Source: Oncotarget. 2017 Dec 14;8(70):115570–81. doi: 10.18632/oncotarget.23311 (PMC5777794; doi:10.18632/oncotarget.23311)
Supplement: Supplementary file 1 [file oncotarget-08-115570-s001.pdf]

Transcriptomic analysis in pediatric spinal ependymoma reveals distinct molecular signatures

SUPPLEMENTARY MATERIALS

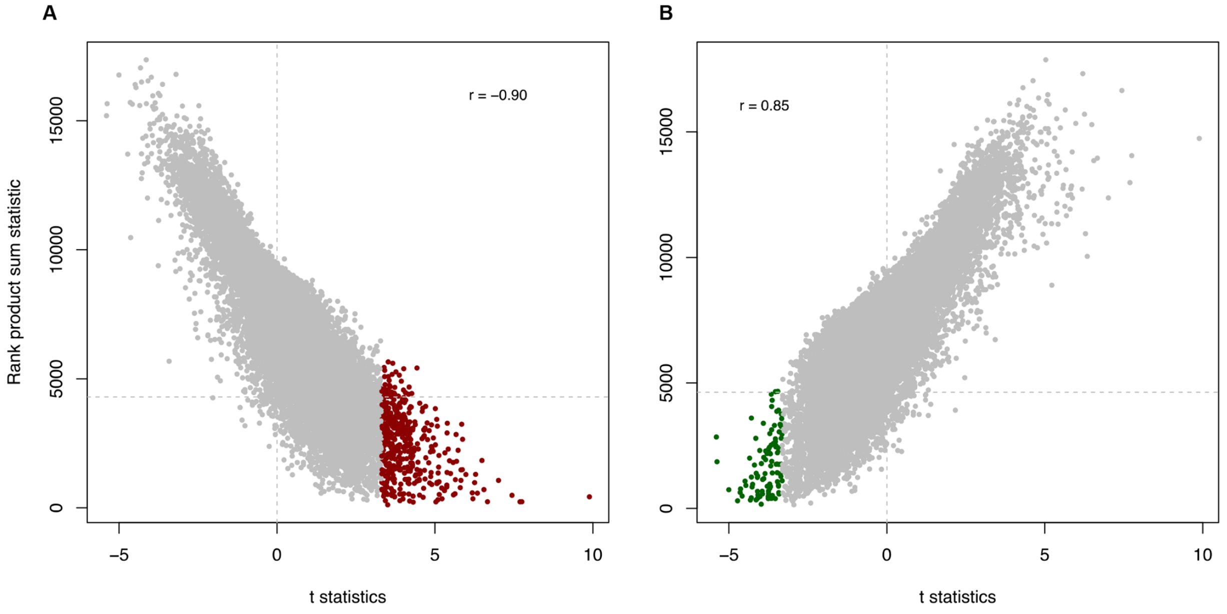

Supplementary Figure 1: Correlation of differential expression between two different methods. Scatter plots of t-statistics (x-axis) from the empirical Bayesian method and estimated rand product sum statistics (y-axis) from the rank product method when **(A)** Pediatric spinal ependymoma (EPN) > intracranial EPN and **(B)** Pediatric spinal EPN < intracranial EPN. Differentially expressed genes were selected at false discovery rate (FDR) < 0.05 using the empirical Bayesian method: up- (red in A) or down- (green in B) regulated genes in pediatric spinal EPN. The dotted horizontal line indicates the top 15% of rank-product sum statistics and the vertical line denotes the zero t-statistics. The correlation between these two statistics was calculated using the Spearman correlation method.

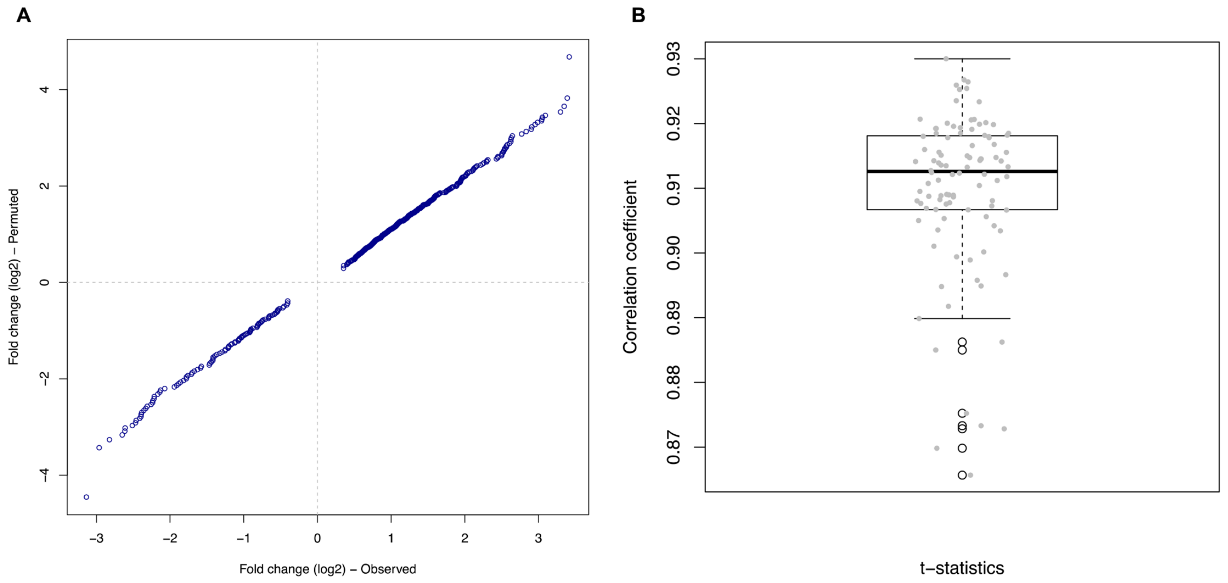

Supplementary Figure 2: Effects of sample imbalance on differential expression. (A) Q-Q plot of fold change (log_2 scale) of 557 differentially expressed genes between pediatric spinal and intracranial ependymomas (EPN) in the original dataset (x-axis) and in permuted datasets (number of permutations = 100) showing similar distributions. **(B)** Box plot of correlation coefficients (Spearman method) calculated from comparing t-statistics of 557 differentially expressed genes in the original dataset with t-statistics from 100 permuted datasets.

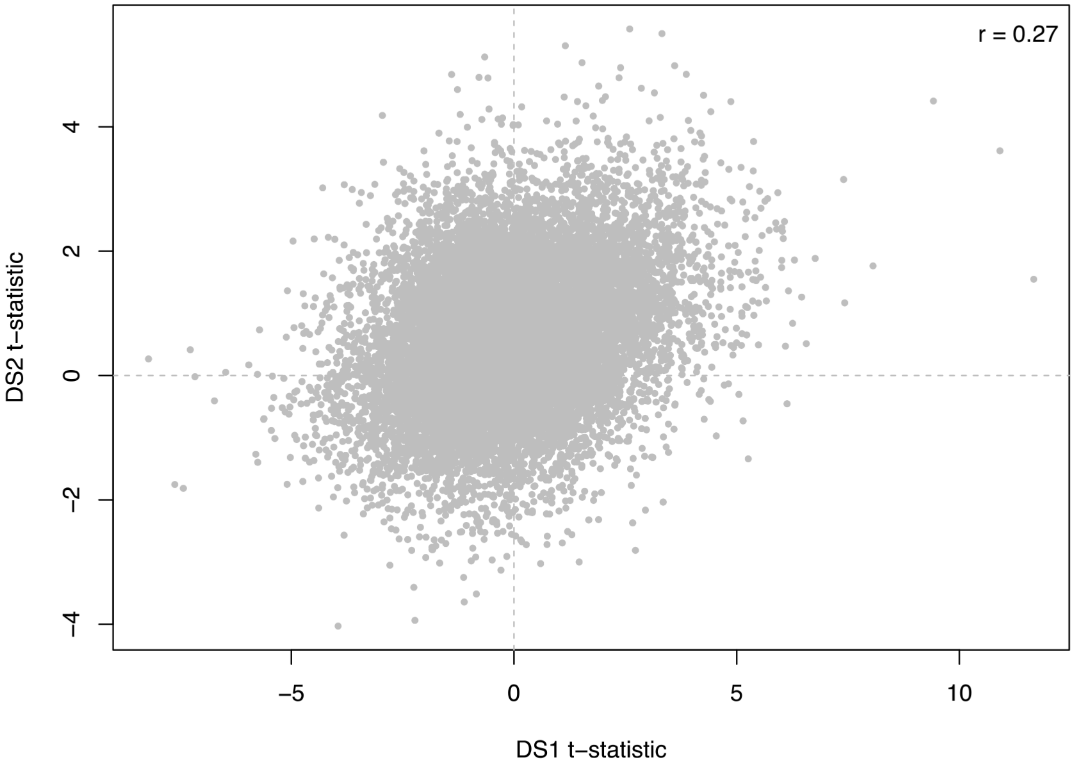

Supplementary Figure 3: Correlations of differential expression between two different datasets. Scatter plot of estimated t-statistics of differential expression for 14,097 genes in dataset 1 (DS1, x-axis) and dataset 2 (DS2, y-axis). The correlation between t-statistics was calculated using the Spearman method.

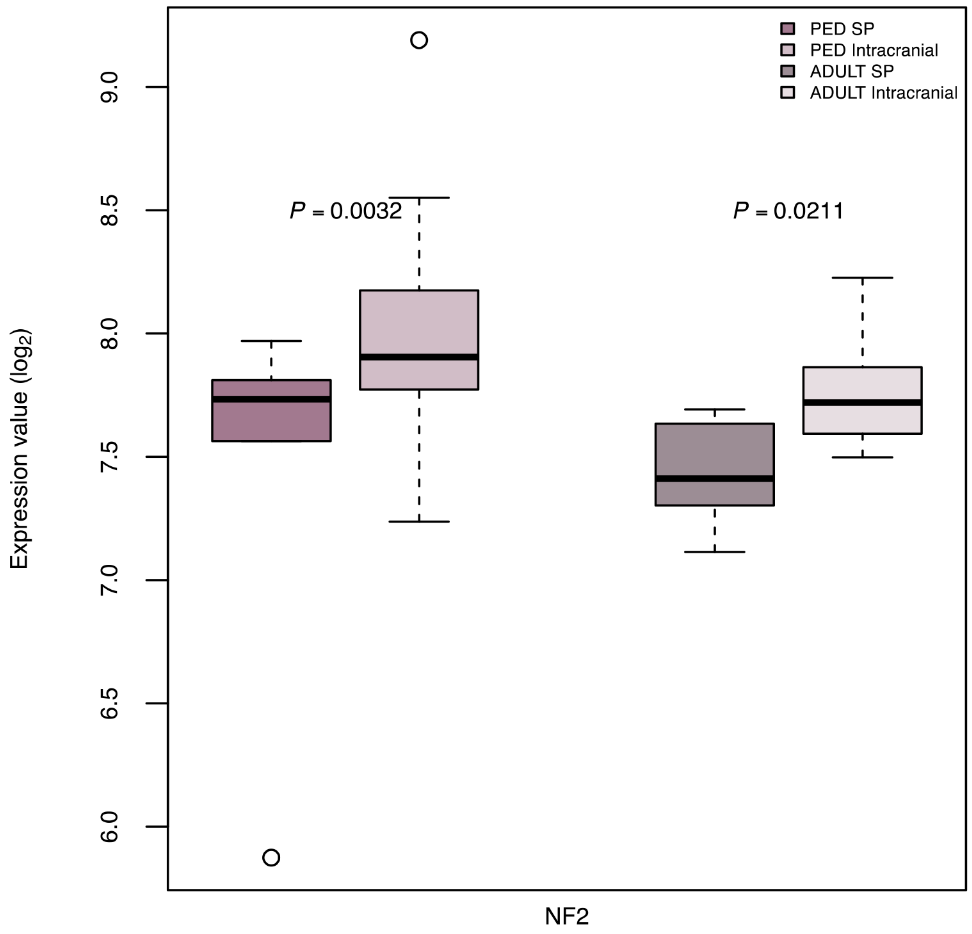

Supplementary Figure 4: NF2 is under-expressed in spinal ependymomas (EPN). Expression level of neurofibromin 2 (NF2) is low in spinal EPN when compared with intracranial EPN both in pediatric (log_2 fold change = −0.51, un-adjusted pvalue = 0.0032) and in adult (log_2 fold change = −0.33, un-adjusted pvalue = 0.0211) patients and was not significant after the multiple testing correction.

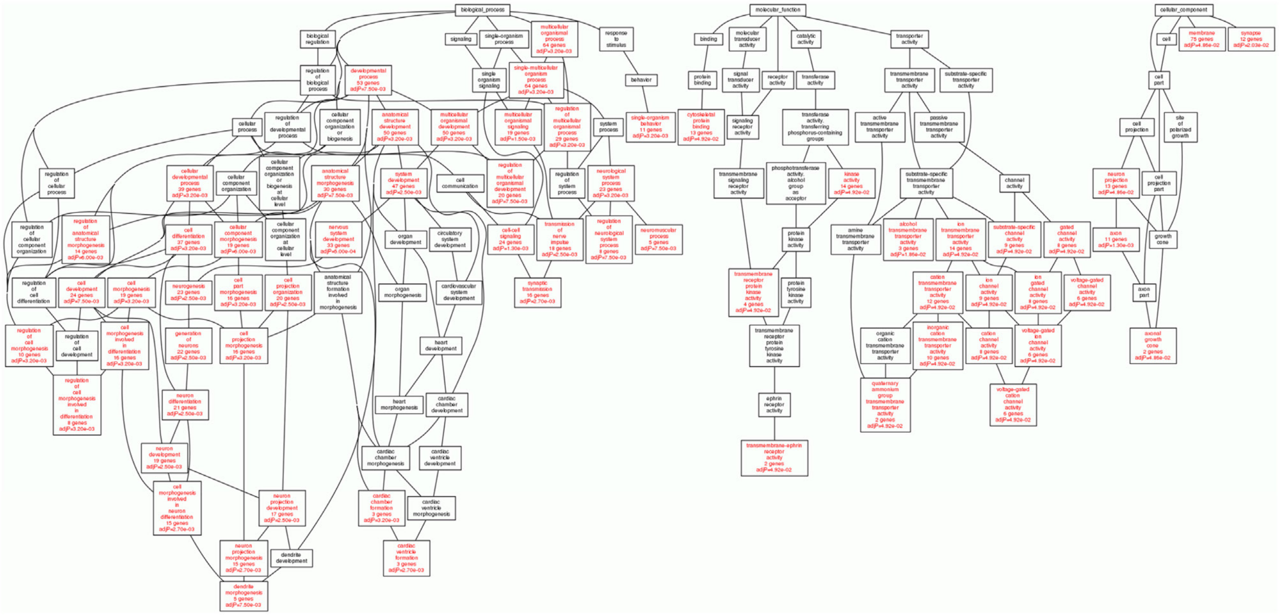

Supplementary Figure 5: Gene ontology (GO) enrichment for miRNA target genes. Enriched directed acyclic graph (DAG) under ‘biological process’, ‘moleuclar function’ and ‘cellular component’ for a set of 159 miRNA target genes. The enriched GO categories are brought together and visualized as a DAG. Categories in red are enriched ones while those in black are non-enriched parents. Listed in the boxes are the name of the GO category, the number of genes in the category and the P-value indicating the significance of enrichment.

Supplementary Table 1: Differential gene expression between pediatric spinal and intracranial ependymomas (EPN). Fold change (logFC on log_2 scale) in expression of 557 genes between pediatric spinal and intracranial EPNs at false discovery rate (FDR) < 0.05 are listed and noted as SP (in Spinal EPN column). Supratentorial EPN indicates whether a gene is up-regulated in pediatric Supratentorial EPN or not (noted as ST, otherwise No) and Posterior Fossa EPN (noted as ST, otherwise No) for up-regulation in pediatric Posterior Fossa EPN.

See Supplementary File 1

Supplementary Table 2: Description of gene expression microarray data sets used in the meta-analysis of pediatric Supratentorial and Posterior fossa ependymomas

Data set	Platform	Data source	N	Reference
1	Affymetrix Exon 1.0ST	GSE27279	75	Witt H (2011)
2	Affymetrix HG-U133 Plus 2	GSE21687	60	Johnson RA (2010)
3	Agilent	GSE27287	40	Witt H (2011)
4	Affymetrix HG-U133 Plus 2	GSE16155	19	Donson AM (2009)
5	Agilent	E-TABM-873	17	Peyre M (2010)
6	Affymetrix HG-U133A 2.0	GSE13267	12	NA

Supplementary Table 3: Enrichment of biological processes

GOID	Category Name	Obs	Exp	ES	FDR
GO:0009952	anterior/posterior pattern specification	17	4.41	3.86	0.001
GO:0009954	proximal/distal pattern formation	7	0.64	11.01	0.001
GO:0007389	pattern specification process	25	9.15	2.73	0.0015
GO:0006119	oxidative phosphorylation	9	1.46	6.16	0.0015
GO:0035113	embryonic appendage morphogenesis	11	2.31	4.76	0.0015
GO:0030326	embryonic limb morphogenesis	11	2.31	4.76	0.0015
GO:0048598	embryonic morphogenesis	26	10.49	2.48	0.0015
GO:0035108	limb morphogenesis	12	2.75	4.36	0.0015
GO:0035107	appendage morphogenesis	12	2.75	4.36	0.0015
GO:0022904	respiratory electron transport chain	11	2.33	4.72	0.0015
GO:0042775	mitochondrial ATP synthesis coupled electron transport	8	1.23	6.51	0.0017
GO:0042773	ATP synthesis coupled electron transport	8	1.23	6.51	0.0017
GO:0045333	cellular respiration	13	3.37	3.86	0.0019
GO:0060173	limb development	12	2.97	4.05	0.0021
GO:0048736	appendage development	12	2.97	4.05	0.0021
GO:0006732	coenzyme metabolic process	15	4.56	3.29	0.0025
GO:0035295	tube development	23	9.24	2.49	0.0025
GO:0022900	electron transport chain	12	3.14	3.83	0.0031
GO:0048706	embryonic skeletal system development	10	2.31	4.33	0.0035
GO:0048705	skeletal system morphogenesis	13	3.79	3.43	0.0035
GO:0051186	cofactor metabolic process	16	5.45	2.94	0.0035
GO:0003002	regionalization	18	6.55	2.75	0.0035
GO:0030534	adult behavior	9	2.03	4.42	0.0067
GO:0006120	mitochondrial electron transport, NADH to ubiquinone	6	0.91	6.59	0.0087
GO:0048704	embryonic skeletal system morphogenesis	8	1.72	4.66	0.0087
GO:0002064	epithelial cell development	8	1.74	4.6	0.0087
GO:0061138	morphogenesis of a branching epithelium	12	3.58	3.35	0.0087
GO:0009653	anatomical structure morphogenesis	65	43.54	1.49	0.0128
GO:0048562	embryonic organ morphogenesis	13	4.6	2.83	0.0168
GO:1901137	carbohydrate derivative biosynthetic process	24	11.8	2.03	0.0182
GO:0009790	embryo development	34	19.07	1.78	0.0182
GO:0003382	epithelial cell morphogenesis	5	0.76	6.55	0.0194

Results of Gene Ontology (GO) enrichment analysis performed on 445 up-regulated genes in pediatric spinal EPN. GO analysis was carried out with biological processes using the WebGestalt 2013 tool and significant GO terms were selected at false discovery rate (FDR) < 0.05. GO identification number (GOID) along with the GO category name (Category Name), observed (Obs) and expected (Exp) genes in each GO category, ratio of enrichment (ES), and the significance of enrichment for the category using Fisher's exact test with for multiple test correction are reported.

Supplementary Table 4: Differential gene expression between adult spinal and intracranial ependymomas (EPN). Fold change (logFC on $\log_2$ scale) in expression of 400 genes between adult spinal and intracranial EPNs at false discovery rate (FDR) < 0.05 are listed. pSEPN indicates whether a gene is up-regulated in pediatric spinal EPN (Yes or No) as reported in Supplementary Table 1

See Supplementary File 1

Supplementary Table 5: Enrichment of chromosomal regions with significantly down-regulated genes in spinal ependymoma (EPN). Enrichment of chromosomal regions by the positional gene enrichment (PGE) method for down-regulated genes in pediatric and adult spinal EPNs at false discovery rate (FDR) < 0.05. The table shows the chromosomal regions: chromosome number (Chr), starting position (Start) end position (End) and the corresposning FDR

See Supplementary File 1

Supplementary Table 6: Putative miRNA–mRNA interactions in pediatric spinal ependymoma (EPN). miRNA-mRNA pairs for the 10 differentially expressed miRNAs in pediatric spinal EPNs were identified from the miRNA/mRNA expression datasets and selected if the association of the pair is significant (FDR < 0.05). These interactions are further required to have predicted target interaction by miRanda (score < −0.5), TargetScan (context score < −0.2) and evolutionary conservation (miRanda conservation score > 0.5). Information on experimentally validated miRNA – mRNA interactions were obtained from the miRTarBase 7.0 database

See Supplementary File 1
