# Supplementary material for: Transcriptomic analysis in pediatric spinal ependymoma reveals distinct molecular signatures
Source: Oncotarget. 2017 Dec 14;8(70):115570–81. doi: 10.18632/oncotarget.23311 (PMC5777794; doi:10.18632/oncotarget.23311)
Supplement: Supplementary file 2 [file oncotarget-08-115570-s002.docx]

**Supplementary Table 1**. **Differential gene expression between pediatric spinal and intracranial ependymomas (EPN)**.

| **Gene** | **logFC** | **FDR** | **SpinalEPN** | **Supratentorial EPN** | **Posterior Fossa**  **EPN** |  |  |  |
| --- | --- | --- | --- | --- | --- | --- | --- | --- |
| HOXA10 | 2.83 | 2.41E-10 | SP | No | No |  |  |  |
| C14orf105 | 3.35 | 6.05E-07 | SP | No | No |  |  |  |
| PRAC1 | 3.39 | 6.05E-07 | SP | No | No |  |  |  |
| HOXB7 | 2.56 | 1.29E-06 | SP | No | No |  |  |  |
| CFTR | 1.94 | 5.92E-06 | SP | No | No |  |  |  |
| SP8 | 3.30 | 2.03E-05 | SP | No | No |  |  |  |
| KMO | 2.21 | 2.75E-05 | SP | No | No |  |  |  |
| NRSN2-AS1 | 1.27 | 3.11E-05 | SP | No | No |  |  |  |
| HOXB13 | 2.58 | 4.92E-05 | SP | No | No |  |  |  |
| MYH2 | 1.76 | 5.57E-05 | SP | No | No |  |  |  |
| ARL15 | 1.89 | 5.73E-05 | SP | No | No |  |  |  |
| DRD1 | 2.90 | 6.52E-05 | SP | No | No |  |  |  |
| KCNJ13 | 2.51 | 6.54E-05 | SP | No | No |  |  |  |
| SYT14 | 1.63 | 0.000135582 | SP | No | No |  |  |  |
| SCGN | 1.02 | 0.000185356 | SP | No | No |  |  |  |
| HOXC9 | 1.54 | 0.000185356 | SP | No | No |  |  |  |
| GLYAT | 0.86 | 0.000185817 | SP | No | No |  |  |  |
| LOXL4 | 1.53 | 0.000185817 | SP | No | No |  |  |  |
| CHEK2 | 1.82 | 0.000185817 | SP | No | No |  |  |  |
| HOXB-AS3 | 2.16 | 0.000192193 | SP | No | No |  |  |  |
| HOXD11 | 1.16 | 0.000239034 | SP | No | No |  |  |  |
| SLC39A2 | 1.40 | 0.000275527 | SP | No | No |  |  |  |
| LOC105377832 | 0.95 | 0.000280347 | SP | No | No |  |  |  |
| LINC00312 | 1.74 | 0.000292507 | SP | No | No |  |  |  |
| CPA3 | 2.54 | 0.000294462 | SP | No | No |  |  |  |
| C20orf85 | 2.62 | 0.000340554 | SP | No | No |  |  |  |
| ARMCX2 | 1.43 | 0.000342197 | SP | No | No |  |  |  |
| CCDC178 | 1.50 | 0.000456906 | SP | No | No |  |  |  |
| MBOAT1 | 1.35 | 0.000499204 | SP | No | No |  |  |  |
| CILP | 1.17 | 0.000544212 | SP | No | No |  |  |  |
| CAPN10 | -0.71 | 0.000544212 | SP | No | No |  |  |  |
| FAM135B | 0.80 | 0.000544212 | SP | No | No |  |  |  |
| VSTM1 | 0.94 | 0.000544212 | SP | No | No |  |  |  |
| HOXA13 | 1.78 | 0.000544212 | SP | No | No |  |  |  |
| HS6ST1 | -1.06 | 0.000544212 | SP | No | No |  |  |  |
| JPH2 | 2.02 | 0.000544212 | SP | No | No |  |  |  |
| PGM5-AS1 | 2.06 | 0.000544212 | SP | No | No |  |  |  |
| CASQ2 | 1.10 | 0.000666389 | SP | No | No |  |  |  |
| EVA1A | 2.14 | 0.000682151 | SP | No | No |  |  |  |
| CYTL1 | 2.91 | 0.000901774 | SP | No | No |  |  |  |
| ADAMTS16 | 2.02 | 0.000905513 | SP | No | No |  |  |  |
| SALL1 | 2.50 | 0.001183134 | SP | No | No | | | |
| HOXC10 | 1.90 | 0.001183134 | SP | No | No | | | |
| WDR31 | 1.23 | 0.001183134 | SP | No | No | | | |
| SRY | 1.45 | 0.001199425 | SP | No | No | | | |
| PCSK5 | 2.04 | 0.001220417 | SP | No | No | | | |
| HOXB6 | 1.65 | 0.001220417 | SP | No | No | | | |
| FRAS1 | 2.95 | 0.00122586 | SP | No | No | | | |
| DIAPH1 | 0.85 | 0.001283957 | SP | No | No | | | |
| CTD-2297D10.2 | 1.95 | 0.001297854 | SP | No | No | | | |
| SIAE | 1.96 | 0.001297854 | SP | No | No | | | |
| TM4SF4 | 0.79 | 0.001304829 | SP | No | No | | | |
| CPNE4 | 3.05 | 0.001412897 | SP | No | No | | | |
| MGST3 | 0.99 | 0.00145386 | SP | No | No | | | |
| ASB17 | 0.72 | 0.00145386 | SP | No | No | | | |
| DPF3 | -1.91 | 0.001483646 | SP | No | No | | | |
| MTRF1 | 0.93 | 0.001772416 | SP | ST | No | | | |
| HMGCS2 | 1.23 | 0.001797516 | SP | No | No | | | |
| FOXE1 | 1.22 | 0.001998774 | SP | No | No | | | |
| ARHGEF28 | 1.99 | 0.002031494 | SP | No | No | | | |
| NAA35 | 0.78 | 0.002043071 | SP | No | No | | | |
| ACSM3 | 1.07 | 0.002043071 | SP | No | No | | |  |
| MIPEP | 1.51 | 0.00209082 | SP | No | No | | |  |
| CHL1-AS2 | 1.75 | 0.002151654 | SP | No | No | | |  |
| FAM175A | 1.18 | 0.002205094 | SP | No | No | | |  |
| HOXD10 | 1.80 | 0.002362203 | SP | No | No | | |  |
| AMDHD1 | 0.87 | 0.002468557 | SP | No | No | | |  |
| LOC100996419 | 1.27 | 0.002556591 | SP | No | No | | |  |
| HOXA11 | 1.02 | 0.002892749 | SP | No | No | | |  |
| SDHD | 0.77 | 0.00298064 | SP | No | No | | |  |
| NALCN | -2.82 | 0.003265656 | SP | No | No | | |  |
| BTBD3 | 1.23 | 0.003397168 | SP | No | No | | |  |
| AP3S1 | 0.64 | 0.0034052 | SP | No | No | | |  |
| ANXA4 | 1.74 | 0.003576446 | SP | No | No | | |  |
| ARL2BP | 0.87 | 0.003576446 | SP | No | No | | |  |
| HOXA7 | 1.59 | 0.003731589 | SP | No | No | | |  |
| ZNF541 | 0.92 | 0.003773163 | SP | No | No | | |  |
| HNF1B | 0.87 | 0.003782569 | SP | No | No | | |  |
| JAKMIP2-AS1 | 0.93 | 0.003879669 | SP | No | No | | |  |
| KIF1A | -2.13 | 0.003879669 | SP | No | No | | |  |
| HOXD8 | 2.15 | 0.003879669 | SP | No | No | | |  |
| INTS6-AS1 | 0.97 | 0.003879669 | SP | No | No | | |  |
| POU3F3 | -2.07 | 0.003948432 | SP | No | No | | |  |
| ANXA3 | 2.45 | 0.003949286 | SP | No | No | | |  |
| MAP7D3 | 0.70 | 0.004088853 | SP | No | No | | |  |
| LOC100270804 | 0.72 | 0.004290217 | SP | No | No | | |  |
| COQ7 | 0.96 | 0.004340058 | SP | No | No | | |  |
| MAPT | -2.38 | 0.004479384 | SP | No | No | | |  |
| LGR4 | 1.56 | 0.005189767 | SP | No | No | | |  |
| DFNB31 | 1.43 | 0.005269307 | SP | No | No | | |  |
| C4orf36 | 0.61 | 0.005390238 | SP | No | No | | |  |
| C21orf62 | 2.52 | 0.005425096 | SP | No | No | | |  |
| TSPAN6 | 1.82 | 0.005481057 | SP | No | No | | |  |
| EPSTI1 | 1.57 | 0.005481057 | SP | No | No | | |  |
| KDELR3 | 1.91 | 0.005481057 | SP | ST | No | | |  |
| ITPK1 | -1.47 | 0.005696924 | SP | No | No | | |  |
| STX17 | 1.07 | 0.005954941 | SP | No | No | | |  |
| SLC51B | 1.33 | 0.006074642 | SP | No | No | | |  |
| SCD | -1.78 | 0.006074642 | SP | No | No | | |  |
| RSPRY1 | 1.03 | 0.006370015 | SP | No | No | | |  |
| SYDE2 | 1.40 | 0.006543716 | SP | No | No | | |  |
| PRICKLE2-AS3 | 0.40 | 0.006543716 | SP | No | No | | |  |
| TEX15 | 1.66 | 0.006543716 | SP | No | No | | |  |
| TMEM64 | 1.58 | 0.007358268 | SP | No | No | | |  |
| PAPD5 | 0.83 | 0.007668502 | SP | No | No | | |  |
| ZDHHC23 | 1.62 | 0.007668502 | SP | No | No | | |  |
| MS4A2 | 0.87 | 0.007947612 | SP | No | No | | |  |
| IGFBP5 | 1.95 | 0.008270889 | SP | No | No | | |  |
| SLC16A5 | 1.06 | 0.008270889 | SP | No | PF | | |  |
| CD82 | -1.03 | 0.008270889 | SP | No | No |  |  |  |
| TMEM261 | 1.08 | 0.008270889 | SP | No | No |  |  |  |
| TUFT1 | 1.19 | 0.008639869 | SP | No | No |  |  |  |
| LSAMP | -2.61 | 0.008825111 | SP | No | No |  |  |  |
| HOXC6 | 3.09 | 0.008951894 | SP | No | No |  |  |  |
| DCDC2 | 2.56 | 0.008951894 | SP | No | No |  |  |  |
| GJA5 | 0.87 | 0.008951894 | SP | No | No |  |  |  |
| PTGR1 | 1.18 | 0.008951894 | SP | No | No |  |  |  |
| CREM | 1.22 | 0.008951894 | SP | No | No |  |  |  |
| RRAGA | 0.82 | 0.008951894 | SP | No | PF |  |  |  |
| SMPDL3A | 1.19 | 0.008951894 | SP | ST | No |  |  |  |
| MYLIP | 1.31 | 0.008951894 | SP | No | No |  |  |  |
| LOC101929480 | 1.27 | 0.008951894 | SP | No | No |  |  |  |
| SCG5 | -2.65 | 0.008951894 | SP | ST | No |  |  |  |
| SLC8B1 | -0.58 | 0.008951894 | SP | No | No |  |  |  |
| ZNF589 | -1.42 | 0.008951894 | SP | ST | No |  |  |  |
| DPYSL3 | 1.40 | 0.009121725 | SP | No | PF |  |  |  |
| LINC01186 | 0.60 | 0.00941959 | SP | No | No |  |  |  |
| APBA2 | -1.88 | 0.00941959 | SP | No | No |  |  |  |
| MRPS6 | 1.23 | 0.009452364 | SP | No | No |  |  |  |
| DHRS11 | 1.10 | 0.009572917 | SP | No | No |  |  |  |
| TOPORS-AS1 | 0.63 | 0.009582774 | SP | No | No |  |  |  |
| TASP1 | 0.87 | 0.009582774 | SP | No | No |  |  |  |
| SLC7A5 | -1.70 | 0.009582774 | SP | No | PF |  |  |  |
| LHX9 | 1.20 | 0.009582774 | SP | No | No |  |  |  |
| KLHL7 | 1.37 | 0.009843147 | SP | No | No |  |  |  |
| CTSL | 0.98 | 0.009843147 | SP | No | No |  |  |  |
| PRUNE | 1.18 | 0.009843147 | SP | No | No |  |  |  |
| IFT22 | 1.01 | 0.009843147 | SP | No | No | |  |  |
| RRAS2 | 1.41 | 0.009843147 | SP | No | No | |  |  |
| EIF3K | 0.64 | 0.009843147 | SP | No | No | |  |  |
| CLDN1 | 1.12 | 0.009943776 | SP | No | No | |  |  |
| C9orf3 | 1.39 | 0.009943776 | SP | No | No | |  |  |
| RFK | 0.94 | 0.010300964 | SP | No | No | |  |  |
| LOC101927943 | 0.74 | 0.010414973 | SP | No | No | |  |  |
| KCNE1 | 2.99 | 0.010501397 | SP | No | No | |  |  |
| PSMB7 | 0.71 | 0.010552824 | SP | No | No | |  |  |
| TOB2 | -1.12 | 0.010552824 | SP | No | No | |  |  |
| IKZF2 | 1.50 | 0.010552824 | SP | No | No | |  |  |
| SLC37A4 | 0.98 | 0.010558743 | SP | No | No | |  |  |
| LOC340184 | 0.52 | 0.01070805 | SP | No | No | |  |  |
| DMKN | 1.83 | 0.010723294 | SP | No | No | |  |  |
| TMEM27 | 0.68 | 0.010723294 | SP | No | No | |  |  |
| PTPN3 | 1.93 | 0.010836719 | SP | No | No | |  |  |
| SHROOM3 | 2.45 | 0.010836719 | SP | No | No | |  |  |
| POP4 | 0.52 | 0.010982011 | SP | No | No | |  |  |
| TOLLIP-AS1 | 0.81 | 0.011019102 | SP | No | No | |  |  |
| GNA14 | 1.12 | 0.011035643 | SP | No | No | |  |  |
| CTSLP8 | 0.87 | 0.011052363 | SP | No | No | |  |  |
| MPDZ | 1.10 | 0.011052363 | SP | No | No | |  |  |
| BAG1 | 0.99 | 0.011052363 | SP | No | No | |  |  |
| SFRP4 | 1.94 | 0.011052363 | SP | No | No | |  |  |
| HEG1 | 1.08 | 0.011052363 | SP | No | No | |  |  |
| CLUL1 | 1.61 | 0.011052363 | SP | No | No | |  |  |
| BAZ2A | -0.75 | 0.011052363 | SP | No | No | |  |  |
| CDIPT-AS1 | 0.76 | 0.011073148 | SP | No | No | |  |  |
| NDUFA8 | 0.80 | 0.011101137 | SP | No | No | |  |  |
| GABRB1 | -2.96 | 0.011101137 | SP | No | No | |  |  |
| TCTN3 | 1.12 | 0.011101137 | SP | No | No | |  |  |
| DTD2 | 1.06 | 0.011101137 | SP | No | No | |  |  |
| APC2 | -1.29 | 0.011101137 | SP | No | No | |  |  |
| TOR1A | 0.86 | 0.011101137 | SP | No | No | |  |  |
| LINC00472 | 1.42 | 0.011101137 | SP | No | No | |  |  |
| FHL1 | 1.89 | 0.011237241 | SP | No | No | |  |  |
| LOC400756 | 0.90 | 0.011510746 | SP | No | No | |  |  |
| TTR | 0.86 | 0.011510746 | SP | No | No | |  |  |
| DENND1A | 1.34 | 0.011885695 | SP | No | No | |  |  |
| PLGRKT | 1.06 | 0.012060941 | SP | No | No | |  |  |
| FAM110B | -1.39 | 0.012075126 | SP | No | No | |  |  |
| KCNA2 | -2.39 | 0.012201616 | SP | No | No | |  |  |
| C14orf132 | -1.46 | 0.012256385 | SP | No | No | |  |  |
| PROSER1 | 1.02 | 0.012256385 | SP | No | No | |  |  |
| STXBP5L | -2.39 | 0.012256385 | SP | No | No | |  |  |
| GNAQ | 1.10 | 0.012386181 | SP | No | No | |  |  |
| MT3 | -2.22 | 0.012386181 | SP | ST | No | |  |  |
| STX5 | 0.67 | 0.012577476 | SP | No | No | |  |  |
| HINT2 | 0.75 | 0.012898641 | SP | No | No | |  |  |
| ATP5O | 0.55 | 0.012978378 | SP | No | No | |  |  |
| CCDC121 | 1.08 | 0.013072676 | SP | No | No | |  |  |
| PGM5 | 1.80 | 0.013222886 | SP | No | No | |  |  |
| TMEM14B | 1.04 | 0.013222886 | SP | No | No | |  |  |
| CTB-12O2.1 | 0.92 | 0.013297038 | SP | No | No | |  |  |
| ATP6V1G1 | 0.79 | 0.013418037 | SP | No | No | |  |  |
| CCDC148 | 1.21 | 0.013418037 | SP | No | No | |  |  |
| CUL5 | 0.88 | 0.013551286 | SP | No | No | |  |  |
| MRPS33 | 0.62 | 0.013551286 | SP | No | No | |  |  |
| LOC101928731 | 1.09 | 0.013551286 | SP | No | No | |  |  |
| CCDC89 | 1.59 | 0.013559376 | SP | No | No | |  |  |
| LMCD1 | 1.39 | 0.013654748 | SP | No | No | |  |  |
| STOML2 | 0.81 | 0.013812533 | SP | No | No | |  |  |
| CACNB1 | -0.88 | 0.013913574 | SP | ST | No | |  |  |
| C1GALT1C1 | 0.90 | 0.013946219 | SP | No | No | |  |  |
| MAGEF1 | 0.59 | 0.013972898 | SP | No | No | |  |  |
| FXYD6 | -2.26 | 0.013972898 | SP | No | No | |  |  |
| COL15A1 | 2.54 | 0.013972898 | SP | No | No | |  |  |
| COQ9 | 0.79 | 0.013972898 | SP | No | No | |  |  |
| VSIG1 | 0.42 | 0.013972898 | SP | No | No | |  |  |
| NDUFB3 | 0.51 | 0.014364265 | SP | No | No | |  |  |
| LYRM1 | 0.65 | 0.014364265 | SP | No | No | |  |  |
| DIXDC1 | 0.99 | 0.014364265 | SP | No | No | |  |  |
| GBAS | 0.78 | 0.014387069 | SP | No | No | |  |  |
| KANK4 | 1.25 | 0.014781569 | SP | No | No | |  |  |
| CHL1 | 3.04 | 0.014797121 | SP | ST | No | |  |  |
| SERPINA3 | -3.13 | 0.015220597 | SP | No | No | |  |  |
| GNG5 | 0.81 | 0.015220597 | SP | No | No | |  |  |
| ACOT8 | 0.82 | 0.015220597 | SP | No | No | |  |  |
| SH3RF3 | -1.18 | 0.015220597 | SP | No | No | |  |  |
| SEPT11 | 1.97 | 0.015220597 | SP | No | No | |  |  |
| CHMP5 | 0.95 | 0.015220597 | SP | No | No | |  |  |
| GOLGA1 | 0.60 | 0.015220597 | SP | No | No | |  |  |
| RNF139-AS1 | 0.74 | 0.016121612 | SP | No | No | |  |  |
| HDHD3 | 0.86 | 0.016121612 | SP | No | No | |  |  |
| FMO1 | 2.63 | 0.016168783 | SP | No | No | |  |  |
| BMPR1B | -1.94 | 0.016168783 | SP | No | No | |  |  |
| PKIA | -2.23 | 0.01656726 | SP | No | No | |  |  |
| CERS3 | 0.51 | 0.016587361 | SP | No | No | |  |  |
| BEGAIN | -1.42 | 0.016809388 | SP | No | No | |  |  |
| MCEE | 0.78 | 0.016823174 | SP | No | No | |  |  |
| FAM69B | 0.86 | 0.016972993 | SP | No | No | |  |  |
| TNMD | 1.97 | 0.017299638 | SP | No | No | |  |  |
| CNTF | 0.90 | 0.017441288 | SP | No | No | |  |  |
| C11orf58 | 0.78 | 0.01755402 | SP | No | No | |  |  |
| DKFZp779M0652 | -0.54 | 0.01755402 | SP | No | No | |  |  |
| SETD3 | 1.32 | 0.01755402 | SP | No | No | |  |  |
| APOO | 0.75 | 0.01755402 | SP | No | No |  |  |  |
| NUDT7 | 1.27 | 0.01755402 | SP | No | No |  |  |  |
| LINC01607 | 1.11 | 0.017633472 | SP | No | No |  |  |  |
| YIPF3 | 0.61 | 0.017633472 | SP | No | No |  |  |  |
| KLHL9 | 0.90 | 0.017633472 | SP | No | No |  |  |  |
| CTSG | 0.63 | 0.017633472 | SP | No | No |  |  |  |
| PCBP2 | -0.99 | 0.017633472 | SP | No | No |  |  |  |
| PLA2G4A | 2.24 | 0.017633472 | SP | No | No |  |  |  |
| TSC22D4 | -1.42 | 0.017633472 | SP | No | No |  |  |  |
| RAB3B | 1.33 | 0.017633472 | SP | No | No |  |  |  |
| FARS2 | 0.81 | 0.017633472 | SP | No | No |  |  |  |
| LRIG3 | 1.43 | 0.017633472 | SP | No | No |  |  |  |
| CXCL12 | 3.05 | 0.017633472 | SP | No | No |  |  |  |
| PKP2 | 2.30 | 0.017858322 | SP | No | No |  |  |  |
| ANGPTL7 | 0.88 | 0.017963809 | SP | No | No |  |  |  |
| AGA | 1.47 | 0.017963809 | SP | No | No |  |  |  |
| CCDC34 | 1.00 | 0.017963809 | SP | No | No |  |  |  |
| RCL1 | 0.87 | 0.018225502 | SP | No | No |  |  |  |
| MIRLET7DHG | 0.45 | 0.018290275 | SP | No | No |  |  |  |
| MAMDC2-AS1 | 0.74 | 0.018290275 | SP | No | No |  |  |  |
| MRPS18C | 0.63 | 0.018291588 | SP | No | No |  |  |  |
| CYGB | 1.52 | 0.018572388 | SP | No | No |  |  |  |
| PRKCQ-AS1 | 1.30 | 0.018581069 | SP | No | No |  |  |  |
| LOC100506473 | 0.62 | 0.018923634 | SP | No | No |  |  |  |
| CASKIN1 | -1.37 | 0.01893578 | SP | No | No |  |  |  |
| FDX1 | 0.73 | 0.01897591 | SP | No | No |  |  |  |
| WWP1 | 0.97 | 0.019224501 | SP | No | No |  |  |  |
| MRPL48 | 0.60 | 0.019521242 | SP | No | No |  |  |  |
| LINC00662 | 1.12 | 0.019533785 | SP | No | No |  |  |  |
| LOC285097 | 1.23 | 0.019543074 | SP | No | No |  |  |  |
| TNPO1 | -1.07 | 0.019566606 | SP | No | No |  |  |  |
| GRSF1 | 0.78 | 0.019566606 | SP | No | No |  |  |  |
| ROBO3 | -1.00 | 0.019566606 | SP | No | No |  |  |  |
| ZBTB6 | 1.16 | 0.019566606 | SP | No | No |  |  |  |
| NEDD1 | 1.11 | 0.01976305 | SP | No | No |  |  |  |
| NMB | 1.55 | 0.019971026 | SP | No | No |  |  |  |
| OXNAD1 | 0.70 | 0.020000952 | SP | No | No |  |  |  |
| CAAP1 | 0.89 | 0.020000952 | SP | No | No |  |  |  |
| ISCA1 | 1.09 | 0.020925863 | SP | No | No |  |  |  |
| SCAMP1-AS1 | 0.67 | 0.021836498 | SP | No | No |  |  |  |
| LOC283861 | 0.73 | 0.021840944 | SP | No | No |  |  |  |
| IL17RA | -0.97 | 0.022003612 | SP | No | No |  |  |  |
| UPRT | 0.70 | 0.022084401 | SP | No | No |  |  |  |
| CBS | -1.78 | 0.022264902 | SP | No | No |  |  |  |
| PRCP | 0.96 | 0.022373089 | SP | No | No |  |  |  |
| VDAC3 | 0.74 | 0.022373089 | SP | No | No |  |  |  |
| STAG3L4 | 0.85 | 0.022585368 | SP | No | No |  |  |  |
| TCF7L2 | 1.28 | 0.023003602 | SP | No | No |  |  |  |
| ATP5J | 0.41 | 0.023289099 | SP | No | No |  |  |  |
| TBC1D19 | 0.77 | 0.023479342 | SP | No | No |  |  |  |
| FH | 1.40 | 0.023731289 | SP | No | No |  |  |  |
| GPM6A | -2.47 | 0.023765901 | SP | No | No |  |  |  |
| SOX11 | -2.41 | 0.023859831 | SP | No | No |  |  |  |
| MTMR12 | 0.77 | 0.023859831 | SP | No | No |  |  |  |
| HOTAIR | 1.59 | 0.024002504 | SP | No | No |  |  |  |
| SCARA3 | -2.14 | 0.024123822 | SP | No | No |  |  |  |
| CACNA1A | -2.24 | 0.024123822 | SP | No | No |  |  |  |
| PSD2 | -2.46 | 0.024123822 | SP | No | No |  |  |  |
| EFNB3 | 1.60 | 0.024431164 | SP | No | No |  |  |  |
| RNF24 | -1.03 | 0.024431164 | SP | No | No |  |  |  |
| IGFBP7 | 1.37 | 0.024438902 | SP | No | No |  |  |  |
| CCDC140 | 0.64 | 0.024467833 | SP | No | No |  |  |  |
| WFDC8 | 0.57 | 0.024670981 | SP | No | No |  |  |  |
| ST20-AS1 | 0.51 | 0.024915273 | SP | No | No |  |  |  |
| PRDX6 | 0.96 | 0.024964813 | SP | No | No |  |  |  |
| HIF3A | -2.31 | 0.024971046 | SP | No | No |  |  |  |
| IGF1 | 2.62 | 0.025131278 | SP | No | No |  |  |  |
| LRRC2 | 2.64 | 0.025131278 | SP | No | No |  |  |  |
| ZNF213-AS1 | 0.61 | 0.025217587 | SP | No | No |  |  |  |
| C7orf61 | -0.90 | 0.02523779 | SP | No | No |  |  |  |
| PDLIM1 | 1.88 | 0.02523779 | SP | ST | No |  |  |  |
| HOXD9 | 0.78 | 0.02523779 | SP | No | No |  |  |  |
| CCDC81 | 2.04 | 0.02523779 | SP | No | No |  |  |  |
| HCCS | 0.80 | 0.02523779 | SP | No | No |  |  |  |
| CCAR2 | 1.02 | 0.025400408 | SP | No | No |  |  |  |
| LOC101928403 | 0.57 | 0.025455371 | SP | No | No |  |  |  |
| FLRT1 | -1.71 | 0.025521275 | SP | No | No |  |  |  |
| MTCP1 | 0.85 | 0.025521275 | SP | No | No |  |  |  |
| FBXO45 | -0.91 | 0.025521275 | SP | No | No |  |  |  |
| SLC18A2 | 1.06 | 0.025521275 | SP | No | No |  |  |  |
| VAPB | -0.77 | 0.025521275 | SP | No | No |  |  |  |
| IL17RB | -1.86 | 0.025521275 | SP | No | No |  |  |  |
| SLC5A3 | 1.13 | 0.025555841 | SP | No | No |  |  |  |
| ANKRD29 | 2.30 | 0.025564739 | SP | No | No |  |  |  |
| NDUFB10 | 0.61 | 0.025564739 | SP | No | No |  |  |  |
| NDUFAB1 | 0.67 | 0.025643316 | SP | No | No |  |  |  |
| SPATA4 | 1.42 | 0.026023038 | SP | No | No |  |  |  |
| SLC6A16 | 1.24 | 0.026023038 | SP | No | No |  |  |  |
| EHBP1 | -1.02 | 0.026345974 | SP | No | No |  |  |  |
| AGTR1 | 0.77 | 0.026345974 | SP | No | No |  |  |  |
| NAV1 | -1.67 | 0.026659706 | SP | No | No |  |  |  |
| IQUB | 1.60 | 0.026681851 | SP | No | No |  |  |  |
| XK | 1.75 | 0.02670326 | SP | No | No |  |  |  |
| ZNF462 | 0.78 | 0.026838233 | SP | No | No |  |  |  |
| PIGO | 0.78 | 0.026838233 | SP | No | No |  |  |  |
| PPAT | -1.20 | 0.026905467 | SP | No | No |  |  |  |
| BMX | 0.36 | 0.027751718 | SP | No | No |  |  |  |
| FAM213A | -0.95 | 0.028101654 | SP | No | No |  |  |  |
| GPT | -0.40 | 0.028101654 | SP | No | No |  |  |  |
| ZNF658 | 1.01 | 0.02829311 | SP | No | No |  |  |  |
| AMER2 | -2.51 | 0.02829311 | SP | No | No |  |  |  |
| ZNF441 | 0.77 | 0.02829311 | SP | No | No |  |  |  |
| XRCC4 | 0.84 | 0.028397427 | SP | No | No |  |  |  |
| NR4A3 | 2.26 | 0.028397427 | SP | No | No |  |  |  |
| RFX3 | 1.65 | 0.028397427 | SP | No | PF |  |  |  |
| ZNF641 | -0.65 | 0.028397427 | SP | No | No |  |  |  |
| MYOF | 1.53 | 0.028397427 | SP | No | No |  |  |  |
| EDDM3A | 0.45 | 0.028397427 | SP | No | No |  |  |  |
| SLC9A5 | -0.47 | 0.028397427 | SP | No | No |  |  |  |
| DLAT | 1.05 | 0.028397427 | SP | No | No |  |  |  |
| RAD54B | -1.12 | 0.028502013 | SP | No | No |  |  |  |
| DHRSX | 0.91 | 0.028607194 | SP | No | No |  |  |  |
| ATP5S | 1.09 | 0.028607194 | SP | No | No |  |  |  |
| SOCS2 | 2.50 | 0.029177369 | SP | No | No |  |  |  |
| WDR44 | 0.66 | 0.029177369 | SP | No | No |  |  |  |
| IKBKE | -0.45 | 0.029294291 | SP | No | No |  |  |  |
| DPM1 | 0.51 | 0.029294291 | SP | No | No |  |  |  |
| LAYN | 1.31 | 0.029294291 | SP | No | No |  |  |  |
| MEGF9 | 1.03 | 0.029294291 | SP | No | No |  |  |  |
| MTSS1L | -1.44 | 0.029294291 | SP | No | No |  |  |  |
| PPP1R26-AS1 | 0.52 | 0.029294291 | SP | No | No |  |  |  |
| HIATL1 | 0.73 | 0.029294291 | SP | No | No |  |  |  |
| SEZ6L | -2.33 | 0.029452431 | SP | ST | No |  |  |  |
| ASXL3 | 2.11 | 0.030037528 | SP | No | No |  |  |  |
| IL18 | 1.43 | 0.030117589 | SP | No | No |  |  |  |
| MMEL1 | 1.04 | 0.03037789 | SP | No | No |  |  |  |
| ZNF396 | 1.21 | 0.030654266 | SP | No | No |  |  |  |
| TBC1D4 | 1.59 | 0.030654266 | SP | No | PF |  |  |  |
| MRPL15 | 0.75 | 0.030843499 | SP | No | No |  |  |  |
| ACOT13 | 0.74 | 0.030935208 | SP | No | No |  |  |  |
| PLA2R1 | 1.44 | 0.031335525 | SP | No | No |  |  |  |
| YPEL5 | 0.56 | 0.031335525 | SP | No | No |  |  |  |
| TLE4 | 1.35 | 0.031364091 | SP | No | No |  |  |  |
| TMEM123 | 0.90 | 0.031628089 | SP | No | PF |  |  |  |
| GSTA4 | -0.82 | 0.031869108 | SP | No | No |  |  |  |
| CMAHP | 1.08 | 0.031869108 | SP | No | No |  |  |  |
| RNF20 | 0.55 | 0.031869108 | SP | No | No |  |  |  |
| NIPSNAP3B | 1.36 | 0.032886705 | SP | No | No |  |  |  |
| ZNF674-AS1 | 0.65 | 0.032886705 | SP | No | No |  |  |  |
| RBMS1 | 1.24 | 0.032886705 | SP | No | No |  |  |  |
| THUMPD1 | 0.75 | 0.033756676 | SP | No | No |  |  |  |
| RIN3 | -0.66 | 0.034323933 | SP | No | No |  |  |  |
| RALY-AS1 | 0.55 | 0.034323933 | SP | No | No |  |  |  |
| PARD6B | 1.37 | 0.034323933 | SP | No | No |  |  |  |
| SEPT9 | -1.26 | 0.034323933 | SP | No | No |  |  |  |
| FGF14-AS2 | 1.35 | 0.034323933 | SP | No | No |  |  |  |
| IFT57 | 1.24 | 0.034323933 | SP | No | PF |  |  |  |
| UBL5 | 0.46 | 0.034512925 | SP | No | No |  |  |  |
| TGFBR2 | 1.49 | 0.034636656 | SP | No | No |  |  |  |
| MRS2 | 0.76 | 0.034861796 | SP | No | No |  |  |  |
| DENND4C | 0.76 | 0.035109492 | SP | No | No |  |  |  |
| ZNF19 | 0.81 | 0.035197053 | SP | No | No |  |  |  |
| RRAGB | 1.04 | 0.035636699 | SP | No | No |  |  |  |
| ZNF200 | 0.66 | 0.035673429 | SP | No | No |  |  |  |
| PPA2 | 1.05 | 0.035720902 | SP | No | No |  |  |  |
| PIGP | 0.78 | 0.035946561 | SP | No | No |  |  |  |
| ABCA8 | -2.35 | 0.035946561 | SP | No | No |  |  |  |
| MLANA | 0.64 | 0.035947818 | SP | No | No |  |  |  |
| DNM1P46 | -0.40 | 0.036041611 | SP | No | No |  |  |  |
| CEP104 | -0.76 | 0.036098074 | SP | No | No |  |  |  |
| SLC2A6 | -0.82 | 0.0361795 | SP | No | No |  |  |  |
| FASTKD5 | 0.42 | 0.036208307 | SP | No | No |  |  |  |
| DUSP14 | 0.88 | 0.036208307 | SP | No | No |  |  |  |
| ZNF480 | 0.35 | 0.036451492 | SP | No | No |  |  |  |
| ITCH | 0.93 | 0.036451492 | SP | No | No |  |  |  |
| STEAP4 | 1.57 | 0.036451492 | SP | No | No |  |  |  |
| AKIP1 | 0.73 | 0.036517142 | SP | No | No |  |  |  |
| UBXN10 | 1.59 | 0.036594922 | SP | No | No |  |  |  |
| ANKRD13B | -0.80 | 0.036594922 | SP | No | No |  |  |  |
| CTGF | 1.72 | 0.036594922 | SP | No | No |  |  |  |
| SEC61B | 0.60 | 0.036594922 | SP | No | No |  |  |  |
| TDP2 | 0.51 | 0.036594922 | SP | No | No |  |  |  |
| PLEKHG4B | 1.28 | 0.036594922 | SP | No | No |  |  |  |
| LRRIQ3 | 0.92 | 0.0366538 | SP | No | No |  |  |  |
| SPEN | -0.91 | 0.036671973 | SP | No | No |  |  |  |
| LRCH1 | 0.78 | 0.036937431 | SP | No | No |  |  |  |
| CDC16 | 0.53 | 0.037008081 | SP | No | No |  |  |  |
| CCDC186 | 0.83 | 0.037008081 | SP | No | No |  |  |  |
| POSTN | 3.42 | 0.037008081 | SP | No | No |  |  |  |
| ADIRF-AS1 | 0.70 | 0.037008081 | SP | No | No |  |  |  |
| PREX1 | -1.07 | 0.037008081 | SP | No | No |  |  |  |
| MAP3K7CL | 1.47 | 0.037008081 | SP | No | No |  |  |  |
| STOML3 | 2.53 | 0.037018727 | SP | No | No |  |  |  |
| DYNC2LI1 | 0.89 | 0.037406697 | SP | No | PF |  |  |  |
| LOC101928433 | 0.86 | 0.037504029 | SP | No | No |  |  |  |
| RIOK1 | 0.64 | 0.037606073 | SP | No | No |  |  |  |
| ATF7IP2 | 0.95 | 0.03761178 | SP | No | No |  |  |  |
| MYL12A | 0.94 | 0.03761178 | SP | No | No |  |  |  |
| NEK3 | 0.75 | 0.038132605 | SP | No | No |  |  |  |
| CFD | 2.01 | 0.038178678 | SP | No | No |  |  |  |
| ZKSCAN3 | 0.62 | 0.038178678 | SP | No | No |  |  |  |
| MIIP | -0.54 | 0.038486618 | SP | No | No |  |  |  |
| BRD7 | 0.68 | 0.038486618 | SP | No | No |  |  |  |
| DCTN3 | 0.75 | 0.038486618 | SP | No | No |  |  |  |
| CHCHD5 | 0.72 | 0.038486618 | SP | No | No |  |  |  |
| AK1 | 1.51 | 0.038486618 | SP | No | No |  |  |  |
| AMT | 0.72 | 0.038486618 | SP | No | No |  |  |  |
| C9orf9 | 1.17 | 0.038486618 | SP | No | PF |  |  |  |
| SLC7A1 | -1.21 | 0.038486618 | SP | No | No |  |  |  |
| C9orf116 | 1.89 | 0.038486618 | SP | No | PF |  |  |  |
| C11orf74 | 0.95 | 0.038486618 | SP | No | No |  |  |  |
| APOBEC3G | 1.33 | 0.038647578 | SP | No | No |  |  |  |
| MKKS | 0.72 | 0.038911932 | SP | No | No |  |  |  |
| HSPB1 | -1.25 | 0.038946973 | SP | No | No |  |  |  |
| LOC286272 | 1.06 | 0.038946973 | SP | No | No |  |  |  |
| GRHPR | 0.64 | 0.039272789 | SP | No | No |  |  |  |
| TMEM14C | 0.53 | 0.039272789 | SP | No | No |  |  |  |
| NCOR2 | -0.92 | 0.039274603 | SP | No | No |  |  |  |
| C2orf72 | -1.63 | 0.039319728 | SP | No | No |  |  |  |
| CBLB | 0.96 | 0.039319728 | SP | No | No |  |  |  |
| MIR31HG | 1.78 | 0.039319728 | SP | No | No |  |  |  |
| CBLL1 | 0.68 | 0.039319728 | SP | No | No |  |  |  |
| ATXN1 | 1.32 | 0.039456683 | SP | No | No |  |  |  |
| JAM3 | 1.36 | 0.040144109 | SP | No | No |  |  |  |
| TTC6 | 2.31 | 0.040249439 | SP | No | No |  |  |  |
| ASIC4 | -0.42 | 0.040249439 | SP | No | No |  |  |  |
| YIF1A | 0.72 | 0.040344906 | SP | No | PF |  |  |  |
| HPGD | 0.96 | 0.040344906 | SP | No | No |  |  |  |
| EPB41L4A | 0.99 | 0.04070505 | SP | No | No |  |  |  |
| IARS2 | 0.79 | 0.040838259 | SP | No | No |  |  |  |
| EID2B | 0.90 | 0.041602274 | SP | No | No |  |  |  |
| URB1-AS1 | 0.78 | 0.041602274 | SP | No | No |  |  |  |
| MTUS1 | 1.66 | 0.04162072 | SP | No | No |  |  |  |
| SLC19A1 | -0.81 | 0.04162072 | SP | No | No |  |  |  |
| SBSPON | 2.12 | 0.04162072 | SP | No | No |  |  |  |
| ATP8A1 | -1.76 | 0.04162072 | SP | No | No |  |  |  |
| ARX | 2.77 | 0.04162072 | SP | No | No |  |  |  |
| CDK7 | 0.64 | 0.04162072 | SP | No | No |  |  |  |
| DSTN | 0.93 | 0.04162072 | SP | No | No |  |  |  |
| SNAP91 | -1.82 | 0.04162072 | SP | ST | No |  |  |  |
| NDUFB5 | 0.56 | 0.04162072 | SP | No | No |  |  |  |
| PSMD5 | 0.96 | 0.04162072 | SP | No | No |  |  |  |
| TTC22 | 0.90 | 0.04162072 | SP | No | No |  |  |  |
| NCKAP5L | -0.52 | 0.041712557 | SP | No | No |  |  |  |
| DLD | 0.91 | 0.041712557 | SP | No | No |  |  |  |
| LINC01158 | -1.06 | 0.041854518 | SP | No | No |  |  |  |
| FGF9 | -2.21 | 0.041854518 | SP | No | No |  |  |  |
| SNRPB2 | 0.50 | 0.041854518 | SP | ST | No |  |  |  |
| PIGC | 0.98 | 0.041898504 | SP | No | No |  |  |  |
| MTMR2 | 0.65 | 0.041898504 | SP | No | No |  |  |  |
| SAR1A | 0.89 | 0.041898504 | SP | No | No |  |  |  |
| ACAT1 | 0.81 | 0.042073079 | SP | No | PF |  |  |  |
| PDE7A | 1.31 | 0.042073079 | SP | No | No |  |  |  |
| WDR38 | 2.09 | 0.042073079 | SP | No | No |  |  |  |
| C16orf46 | 0.92 | 0.042073079 | SP | No | No |  |  |  |
| ITPK1-AS1 | -1.16 | 0.042073079 | SP | No | No |  |  |  |
| CMC1 | 0.61 | 0.042110611 | SP | No | No |  |  |  |
| LHX2 | -2.17 | 0.042232015 | SP | No | No |  |  |  |
| CDH17 | 0.48 | 0.042410343 | SP | No | No |  |  |  |
| RXRA | -0.90 | 0.042410343 | SP | No | No |  |  |  |
| TECR | -0.66 | 0.042527325 | SP | No | No |  |  |  |
| MOCS3 | 0.65 | 0.042527325 | SP | No | No |  |  |  |
| SLC12A3 | 0.46 | 0.042527325 | SP | No | No |  |  |  |
| UQCC1 | 0.62 | 0.042615945 | SP | No | No |  |  |  |
| TRIM45 | 0.86 | 0.042779646 | SP | No | No |  |  |  |
| TMEM185B | 0.88 | 0.043046631 | SP | No | No |  |  |  |
| STAU1 | 0.70 | 0.043126131 | SP | No | No |  |  |  |
| HOXA11-AS | 0.75 | 0.043131345 | SP | No | No |  |  |  |
| ELMOD2 | 1.21 | 0.04321202 | SP | No | No |  |  |  |
| FOXG1 | -2.61 | 0.04321202 | SP | No | No |  |  |  |
| RGS13 | 1.08 | 0.04329256 | SP | No | No |  |  |  |
| SIRT5 | 0.54 | 0.043498398 | SP | No | No |  |  |  |
| GMCL1 | 0.70 | 0.043499372 | SP | No | No |  |  |  |
| MATN2 | 2.43 | 0.04350397 | SP | No | No |  |  |  |
| LINC00908 | 0.42 | 0.043695077 | SP | No | No |  |  |  |
| ZNF474 | 1.53 | 0.044280732 | SP | No | No |  |  |  |
| LOC100287896 | 0.63 | 0.044280732 | SP | No | No |  |  |  |
| ATP5H | 0.49 | 0.044443596 | SP | No | No |  |  |  |
| RTP4 | 1.05 | 0.044584405 | SP | No | No |  |  |  |
| SLK | 0.67 | 0.044651229 | SP | No | No |  |  |  |
| COL20A1 | -0.57 | 0.044712446 | SP | No | No |  |  |  |
| FAM104B | 0.71 | 0.044875148 | SP | No | No |  |  |  |
| LOC101929132 | 0.75 | 0.044875148 | SP | No | No |  |  |  |
| CD24 | 2.61 | 0.044881666 | SP | No | No |  |  |  |
| FERMT1 | -1.33 | 0.04490473 | SP | No | No |  |  |  |
| SLC52A3 | -0.61 | 0.045177853 | SP | No | No |  |  |  |
| CADM4 | 1.07 | 0.045177853 | SP | No | No |  |  |  |
| MDM1 | 1.23 | 0.045177853 | SP | No | No |  |  |  |
| FAM76A | 1.07 | 0.045177853 | SP | No | No |  |  |  |
| NT5E | 1.94 | 0.045411518 | SP | No | No |  |  |  |
| DCK | 0.86 | 0.045411518 | SP | No | No |  |  |  |
| GK | 1.04 | 0.045505135 | SP | No | No |  |  |  |
| CTBS | 1.08 | 0.045505135 | SP | No | No |  |  |  |
| LINC01091 | 1.50 | 0.045918761 | SP | No | No |  |  |  |
| LINC01549 | 0.76 | 0.04594259 | SP | No | No |  |  |  |
| MUC12 | 0.74 | 0.046730545 | SP | No | No |  |  |  |
| AK4 | -1.58 | 0.046886243 | SP | No | No |  |  |  |
| GIT1 | -0.66 | 0.047874563 | SP | No | No |  |  |  |
| CAMK2D | -1.21 | 0.047998258 | SP | No | No |  |  |  |
| UBE2I | -0.80 | 0.047998258 | SP | No | No |  |  |  |
| BRD2 | -0.54 | 0.048399196 | SP | No | No |  |  |  |
| CRADD | 0.66 | 0.048744422 | SP | No | No |  |  |  |
| POT1 | 0.97 | 0.04879031 | SP | No | No |  |  |  |
| GTPBP8 | 0.60 | 0.04879031 | SP | No | No |  |  |  |
| MRPL50 | 1.14 | 0.04879031 | SP | No | No |  |  |  |
| RPAP3 | 0.87 | 0.04879031 | SP | No | No |  |  |  |
| CD58 | 1.25 | 0.04879031 | SP | No | No |  |  |  |
| KATNAL2 | 1.22 | 0.04879031 | SP | No | No |  |  |  |
| DNASE1L3 | 0.76 | 0.049131095 | SP | No | No |  |  |  |
| TDRD7 | 0.93 | 0.049226218 | SP | No | No |  |  |  |
| GPATCH8 | -0.56 | 0.049226218 | SP | No | No |  |  |  |
| CCT6B | 0.77 | 0.049226218 | SP | No | No |  |  |  |
| PRORSD1P | 0.57 | 0.049226218 | SP | No | No |  |  |  |
| WT1 | 1.56 | 0.049226218 | SP | No | No |  |  |  |
| ZNF382 | 0.65 | 0.049301322 | SP | No | No |  |  |  |
| HOXB8 | 0.74 | 0.049316286 | SP | No | No |  |  |  |
| SNX31 | 1.84 | 0.049316286 | SP | No | No |  |  |  |
| LOXL1-AS1 | 1.18 | 0.049316286 | SP | No | No |  |  |  |
| SLC25A21-AS1 | 0.57 | 0.049316286 | SP | No | No |  |  |  |
| PRSS23 | 2.12 | 0.049316286 | SP | No | No |  |  |  |
| TRDN | 1.51 | 0.049316286 | SP | No | No |  |  |  |
| KLHDC7A | 0.69 | 0.049316286 | SP | No | No |  |  |  |
| ERGIC3 | 0.84 | 0.049350045 | SP | No | No |  |  |  |
| ZFAND5 | 1.18 | 0.049552902 | SP | No | PF |  |  |  |
| EP400 | -0.74 | 0.049563261 | SP | No | No |  |  |  |
| RAB11FIP1 | 1.64 | 0.049582231 | SP | No | No |  |  |  |
| ATRAID | 0.58 | 0.049739655 | SP | No | No |  |  |  |
| CSTF3 | 0.83 | 0.049739655 | SP | No | No |  |  |  |
| MSANTD3 | 0.88 | 0.049739655 | SP | No | No |  |  |  |
| FBXO16 | 0.84 | 0.049786407 | SP | No | No |  |  |  |
| COPB1 | 0.55 | 0.049889479 | SP | No | No |  |  |  |
| TMEM60 | 0.55 | 0.049913974 | SP | No | No |  |  |  |
| LIN7A | -1.59 | 0.049913974 | SP | No | No |  |  |  |
| DUSP11 | 0.53 | 0.049913974 | SP | No | No |  |  |  |

**Supplementary Table 4**. **Differential gene expression between adult spinal and intracranial ependymomas (EPN)**.

| **Gene** | **logFC** | **FDR** | **pSEPN** |
| --- | --- | --- | --- |
| COL22A1 | 3.49 | 5.94E-05 | No |
| JPH2 | 3.15 | 7.73E-05 | Yes |
| LOC389895 | 1.85 | 0.000170939 | No |
| HOXB8 | 1.84 | 0.000298791 | Yes |
| FZD2 | -2.32 | 0.001506322 | No |
| CILP | 1.85 | 0.001573935 | Yes |
| ZNF483 | 1.83 | 0.002807248 | No |
| NLK | 1.18 | 0.002807248 | No |
| ZIC1 | -5.43 | 0.002807248 | No |
| DDX39A | -1.00 | 0.003215992 | No |
| PRRX1 | -3.48 | 0.003501332 | No |
| ARHGEF28 | 2.35 | 0.003501332 | Yes |
| CTTNBP2 | 2.82 | 0.005308943 | No |
| SLC35F4 | 1.39 | 0.005308943 | No |
| ZNF275 | 1.04 | 0.005308943 | No |
| SLC16A10 | -2.71 | 0.005361136 | No |
| PI16 | 1.58 | 0.006752768 | No |
| BGN | -2.95 | 0.007343462 | No |
| B3GAT2 | 3.40 | 0.007343462 | No |
| COX11 | 1.01 | 0.008895828 | No |
| LOC440934 | 1.16 | 0.008895828 | No |
| CRTC3 | 0.83 | 0.008895828 | No |
| LOC283861 | 1.17 | 0.008895828 | Yes |
| GALK2 | 1.22 | 0.009623787 | No |
| TMOD1 | 2.87 | 0.009623787 | No |
| CDYL | 1.14 | 0.009623787 | No |
| HPSE2 | 1.97 | 0.009623787 | No |
| CXorf40B | 0.80 | 0.009623787 | No |
| DNAJC15 | 1.28 | 0.009623787 | No |
| LINC00515 | 1.39 | 0.009623787 | No |
| NECAB2 | 1.03 | 0.009623787 | No |
| CELF6 | 1.57 | 0.009623787 | No |
| MLLT6 | 0.74 | 0.009623787 | No |
| EFNB3 | 2.11 | 0.009623787 | Yes |
| SLC1A5 | -1.03 | 0.010292299 | No |
| PWWP2A | 0.88 | 0.010292299 | No |
| MIR31HG | 3.74 | 0.010420263 | Yes |
| CFTR | 4.40 | 0.010720417 | Yes |
| CXorf40A | 0.83 | 0.010833325 | No |
| MECOM | -3.29 | 0.012140826 | No |
| CNTFR-AS1 | 0.48 | 0.012140826 | No |
| ZIC2 | -5.12 | 0.012519721 | No |
| RASSF3 | -1.89 | 0.012519721 | No |
| DYNC1LI2 | 0.67 | 0.012692894 | No |
| NCOR2 | -1.05 | 0.012933084 | Yes |
| RANGRF | 1.01 | 0.013050437 | No |
| CHEK2 | 2.55 | 0.013682483 | Yes |
| SGPP2 | 3.53 | 0.013762105 | No |
| GPR161 | -2.20 | 0.013876809 | No |
| ATL3 | -1.47 | 0.013876809 | No |
| HOXB-AS3 | 1.70 | 0.014192445 | Yes |
| SOD1 | 0.89 | 0.014465883 | No |
| GCSH | 1.10 | 0.014473983 | No |
| UNC50 | 0.75 | 0.014518318 | No |
| GMNC | 1.34 | 0.014518318 | No |
| GNB5 | 0.82 | 0.014518318 | No |
| MTFMT | 0.97 | 0.014518318 | No |
| SPAG9 | 0.90 | 0.014518318 | No |
| KAT8 | 0.77 | 0.014518318 | No |
| TMEM27 | 1.16 | 0.014518318 | Yes |
| ACTR3C | 1.14 | 0.014936092 | No |
| PDHA1 | 0.89 | 0.014936092 | No |
| RGS19 | -1.29 | 0.014936092 | No |
| MFSD6 | 1.85 | 0.014936092 | No |
| C6orf89 | 0.62 | 0.014936092 | No |
| CD300LG | 1.05 | 0.014936092 | No |
| ARMCX3 | 1.21 | 0.014936092 | No |
| NDUFA5 | 0.64 | 0.014936092 | No |
| TRIM16 | 1.79 | 0.014936092 | No |
| PPP2R1B | -0.94 | 0.014936092 | No |
| FBLN1 | -3.13 | 0.014936092 | No |
| FKBP14 | -1.08 | 0.014936092 | No |
| HOXA7 | 3.06 | 0.014936092 | Yes |
| COQ7 | 1.00 | 0.014936092 | Yes |
| CACNA1A | -2.95 | 0.014936092 | Yes |
| CLEC16A | 0.70 | 0.016740177 | No |
| HAS2 | 2.99 | 0.0167479 | No |
| MORF4L2-AS1 | 0.51 | 0.016964495 | No |
| MXRA7 | 0.86 | 0.017076764 | No |
| LDOC1 | 1.55 | 0.017076764 | No |
| LRCH2 | 2.52 | 0.017517106 | No |
| RIMS3 | 0.93 | 0.017698918 | No |
| TIMM8A | 0.90 | 0.017698918 | No |
| DNAJA2 | 0.72 | 0.017818435 | No |
| EIF3J-AS1 | 0.95 | 0.017818435 | No |
| ARPC1B | -1.49 | 0.017818435 | No |
| ATXN1 | 1.13 | 0.017818435 | Yes |
| CCT6B | 1.57 | 0.017818435 | Yes |
| RASD2 | -0.77 | 0.018610468 | No |
| USP22 | 0.85 | 0.018688384 | No |
| MRPL2 | 0.78 | 0.018893506 | No |
| FAM120C | 1.33 | 0.018893506 | No |
| STX6 | -0.88 | 0.018893506 | No |
| NDNL2 | 0.84 | 0.018911519 | No |
| PLD6 | 1.25 | 0.018911519 | No |
| CCDC109B | -1.81 | 0.018911519 | No |
| PCYOX1 | 0.87 | 0.018911519 | No |
| FAM135B | 1.04 | 0.018911519 | Yes |
| FXN | 1.00 | 0.018981507 | No |
| WBSCR17 | 1.95 | 0.018981507 | No |
| DCAF6 | -1.14 | 0.018981507 | No |
| PUSL1 | -0.63 | 0.018997584 | No |
| PRKCD | -1.12 | 0.019567811 | No |
| PIGL | 1.08 | 0.019567811 | No |
| DPH6-AS1 | 1.71 | 0.019567811 | No |
| AHNAK2 | -2.25 | 0.019567811 | No |
| FAM227B | 1.00 | 0.019567811 | No |
| THUMPD1 | 0.90 | 0.019567811 | Yes |
| ACOT9 | 0.69 | 0.019714113 | No |
| CASP6 | -1.54 | 0.019815062 | No |
| FNBP1 | -1.25 | 0.019996685 | No |
| ZNF674-AS1 | 1.26 | 0.020077649 | Yes |
| MAP7D3 | 1.01 | 0.020195815 | Yes |
| CWC25 | 0.60 | 0.020330122 | No |
| MTCH1 | 0.68 | 0.020356594 | No |
| SHC4 | 2.84 | 0.021339797 | No |
| HSBP1 | 1.06 | 0.021339797 | No |
| ANKRD13D | -1.00 | 0.021339797 | No |
| MPP3 | 0.99 | 0.021339797 | No |
| ST3GAL4 | -0.94 | 0.021641577 | No |
| REEP4 | -0.82 | 0.021700689 | No |
| SCN1A | 3.52 | 0.0217813 | No |
| C15orf40 | 0.77 | 0.021975862 | No |
| BAZ1A | -1.35 | 0.021975862 | No |
| YY1AP1 | -0.59 | 0.021975862 | No |
| TIMM21 | 0.69 | 0.022673476 | No |
| PIK3R3 | -2.13 | 0.022673476 | No |
| MKNK2 | -1.31 | 0.022673476 | No |
| FAM192A | 0.67 | 0.022673476 | No |
| VWA9 | 0.61 | 0.022673476 | No |
| WIPF2 | 0.68 | 0.022673476 | No |
| HOXB7 | 2.78 | 0.022712222 | Yes |
| SLC27A6 | 4.02 | 0.022762437 | No |
| PDCD4 | 0.94 | 0.022762437 | No |
| RRAGB | 1.22 | 0.022762437 | Yes |
| SNX31 | 2.38 | 0.022762437 | Yes |
| EMC8 | 0.64 | 0.023147747 | No |
| FUNDC2 | 0.87 | 0.023663213 | No |
| LOC441086 | 1.12 | 0.023663213 | No |
| RAB9A | 1.20 | 0.02378503 | No |
| SNX1 | 0.77 | 0.02378503 | No |
| ASH1L | -0.48 | 0.025450956 | No |
| FAM228A | 0.81 | 0.025493908 | No |
| SELO | -0.58 | 0.025903653 | No |
| INPPL1 | -0.57 | 0.026073989 | No |
| MARCKSL1 | -1.67 | 0.026394477 | No |
| EID1 | 0.88 | 0.026394477 | No |
| CLMN | -2.14 | 0.026394477 | No |
| AREL1 | -0.62 | 0.026394477 | No |
| KRBA2 | 0.95 | 0.026394477 | No |
| STAT2 | -0.77 | 0.026394477 | No |
| ZNF788 | -0.63 | 0.026394477 | No |
| PCLO | 1.90 | 0.026394477 | No |
| APBA2 | -2.43 | 0.026394477 | Yes |
| EHBP1L1 | -1.13 | 0.02686658 | No |
| PPARGC1A | 2.66 | 0.027278181 | No |
| CMC4 | 1.54 | 0.027278181 | No |
| TRMT1 | -0.78 | 0.027278181 | No |
| SYNDIG1 | -2.63 | 0.027478812 | No |
| CERK | -1.79 | 0.027478812 | No |
| CCDC178 | 1.38 | 0.02760644 | Yes |
| EPB41L3 | 2.06 | 0.027868665 | No |
| RABEPK | 0.56 | 0.027868665 | No |
| RBBP7 | 0.67 | 0.027868665 | No |
| RGMA | 1.79 | 0.027868665 | No |
| CALHM2 | -1.30 | 0.027868665 | No |
| TUFM | 0.72 | 0.028023065 | No |
| BCAR3 | 1.07 | 0.028023065 | No |
| AKTIP | 0.79 | 0.028023065 | No |
| TGFB3 | -1.15 | 0.028023065 | No |
| TUBG2 | 0.97 | 0.028237191 | No |
| CPEB1 | 2.08 | 0.030305091 | No |
| EVA1C | 2.26 | 0.03050166 | No |
| TICAM1 | -0.67 | 0.03050166 | No |
| GFOD1 | -1.05 | 0.03050166 | No |
| ZIC4 | -0.90 | 0.03050166 | No |
| USP27X-AS1 | 0.86 | 0.03082028 | No |
| CA5B | 1.52 | 0.03115063 | No |
| GABARAPL2 | 1.04 | 0.03152861 | No |
| ACTR3 | -0.86 | 0.03152861 | No |
| ARL2BP | 0.89 | 0.03152861 | Yes |
| PHB | 0.65 | 0.032383962 | No |
| LOC100128288 | 0.80 | 0.032497883 | No |
| SCRN2 | 0.79 | 0.032677825 | No |
| BMP4 | -1.47 | 0.032677825 | No |
| PSTPIP2 | -1.31 | 0.032850592 | No |
| EMID1 | -1.51 | 0.033382584 | No |
| TCEAL3 | 1.33 | 0.033382584 | No |
| TMEM109 | -0.76 | 0.033382584 | No |
| MB21D2 | -1.45 | 0.033382584 | No |
| MRPL39 | 0.71 | 0.033382584 | No |
| NDUFB10 | 0.70 | 0.033382584 | Yes |
| SNED1 | -2.63 | 0.033886567 | No |
| CRTC2 | -0.56 | 0.034157889 | No |
| ZNF319 | 0.71 | 0.034966024 | No |
| RABL6 | -0.59 | 0.035390089 | No |
| NGLY1 | -0.65 | 0.035390089 | No |
| JAM2 | 2.45 | 0.035390089 | No |
| DGCR2 | -0.66 | 0.035412184 | No |
| DIRAS2 | 2.89 | 0.035690505 | No |
| LYN | -1.79 | 0.035690505 | No |
| ZNF800 | 0.96 | 0.035690505 | No |
| CDR1 | 2.71 | 0.035690505 | No |
| SLC7A5 | -2.46 | 0.035690505 | Yes |
| FAM96B | 0.75 | 0.035814902 | No |
| PARP16 | 0.62 | 0.035814902 | No |
| KIF27 | 1.14 | 0.035870601 | No |
| METTL23 | 0.71 | 0.035870601 | No |
| VWA5A | 1.41 | 0.035870601 | No |
| TMEM135 | -0.84 | 0.035870601 | No |
| HOXB6 | 2.07 | 0.035870601 | Yes |
| TSPAN6 | 1.67 | 0.035870601 | Yes |
| IMMP2L | 0.74 | 0.036026876 | No |
| TMCO6 | 0.84 | 0.036065462 | No |
| TCEANC | 0.79 | 0.036065462 | No |
| UAP1L1 | -1.24 | 0.036068607 | No |
| CTDSP2 | -0.81 | 0.03632417 | No |
| SEPT9 | -1.08 | 0.03632417 | Yes |
| MED15 | -0.63 | 0.036415834 | No |
| ZKSCAN8 | 0.79 | 0.036750086 | No |
| SACS | -0.75 | 0.036750086 | No |
| LOC285812 | -1.59 | 0.036750086 | No |
| YEATS2 | -0.85 | 0.036773494 | No |
| ECI2 | 0.93 | 0.036819773 | No |
| XKR4 | 2.86 | 0.036819773 | No |
| DNAJC9-AS1 | 0.89 | 0.036819773 | No |
| LIFR | 1.93 | 0.036819773 | No |
| DOT1L | -0.84 | 0.036819773 | No |
| CARS | -0.89 | 0.036819773 | No |
| IFT20 | 0.76 | 0.037016585 | No |
| RBM26-AS1 | 1.03 | 0.037033526 | No |
| ZNF276 | -0.45 | 0.037076496 | No |
| AMOT | 0.68 | 0.037076496 | No |
| LOC400756 | 1.14 | 0.037076496 | Yes |
| USP11 | 0.88 | 0.037088289 | No |
| IKBKE | -0.47 | 0.037088289 | Yes |
| DAP3 | -0.48 | 0.037432488 | No |
| CHST9 | 3.37 | 0.037624855 | No |
| ELP5 | 0.61 | 0.037764622 | No |
| ASAP1-IT1 | -0.94 | 0.038094559 | No |
| MIRLET7BHG | -1.02 | 0.038291803 | No |
| PPP1R9A | 1.83 | 0.038291803 | No |
| FAM89B | -0.73 | 0.038314969 | No |
| CD24 | 2.23 | 0.038314969 | Yes |
| ZFAND2B | -0.51 | 0.03875447 | No |
| GLUD2 | 1.04 | 0.03875447 | No |
| SNRNP25 | 0.79 | 0.038784264 | No |
| CTGF | 2.37 | 0.038784264 | Yes |
| RPE65 | 2.80 | 0.038929507 | No |
| INHBA | 2.70 | 0.039064139 | No |
| VWA1 | -1.52 | 0.039064139 | No |
| KCNE4 | -2.54 | 0.039064139 | No |
| PDXK | -0.79 | 0.039304888 | No |
| NDUFB6 | 0.57 | 0.039304888 | No |
| C21orf2 | 0.70 | 0.039304888 | No |
| CYP11A1 | 1.28 | 0.039304888 | No |
| LOC100287590 | 0.92 | 0.039304888 | No |
| MTMR10 | 0.73 | 0.039304888 | No |
| COLGALT1 | -0.92 | 0.039304888 | No |
| C1GALT1C1 | 1.05 | 0.039304888 | Yes |
| BRWD1 | 1.09 | 0.03932532 | No |
| GLIPR2 | 1.51 | 0.039377748 | No |
| DIRC3 | 1.46 | 0.039739291 | No |
| RAB40AL | 0.70 | 0.03974277 | No |
| HOXC6 | 3.90 | 0.03974277 | Yes |
| C17orf75 | 0.75 | 0.040128843 | No |
| ALPK1 | -0.34 | 0.040128843 | No |
| MCF2L | -1.43 | 0.040128843 | No |
| ARF5 | 0.55 | 0.040128843 | No |
| DAB1 | 2.71 | 0.040128843 | No |
| TRAF5 | -1.91 | 0.04042659 | No |
| ZNF385B | 1.91 | 0.040555131 | No |
| OSER1-AS1 | 0.83 | 0.040555131 | No |
| HMHA1 | -1.32 | 0.040555131 | No |
| EPB41L5 | 1.23 | 0.040574057 | No |
| ASTN1 | 3.21 | 0.040585995 | No |
| C6orf62 | 0.77 | 0.040758567 | No |
| BIN1 | -1.35 | 0.040758567 | No |
| GKAP1 | 1.06 | 0.040758567 | No |
| LINC00526 | 0.70 | 0.040758567 | No |
| PNPLA4 | 1.26 | 0.040758567 | No |
| C7orf31 | 1.47 | 0.040758567 | No |
| LMBRD1 | 1.08 | 0.040758567 | No |
| CCDC71L | 1.62 | 0.040758567 | No |
| DCUN1D5 | -0.75 | 0.040758567 | No |
| IFITM1 | -2.99 | 0.040758567 | No |
| MOV10 | -1.13 | 0.040758567 | No |
| HOXC9 | 2.51 | 0.040758567 | Yes |
| CPNE4 | 3.99 | 0.040758567 | Yes |
| UVRAG | -0.57 | 0.041209969 | No |
| VARS | -0.59 | 0.041619858 | No |
| GDA | -3.08 | 0.041619858 | No |
| HDGF | -0.79 | 0.041619858 | No |
| YPEL1 | -0.89 | 0.041866502 | No |
| HSPA13 | 0.90 | 0.042086817 | No |
| C21orf62 | 2.37 | 0.04216085 | Yes |
| PPM1F | -0.91 | 0.042382709 | No |
| FBXO46 | -0.56 | 0.042420964 | No |
| TNFAIP1 | 0.76 | 0.042589632 | No |
| PHRF1 | -0.84 | 0.042710149 | No |
| ZNF808 | 0.99 | 0.042710149 | No |
| LRRC2 | 2.25 | 0.042710149 | Yes |
| PYROXD2 | 1.26 | 0.042855067 | No |
| TSN | 0.51 | 0.042855067 | No |
| AIDA | -0.52 | 0.042855067 | No |
| HN1 | -1.20 | 0.042855067 | No |
| GNPNAT1 | -0.99 | 0.042855067 | No |
| ERI2 | 0.98 | 0.042870283 | No |
| RBM11 | 1.16 | 0.043686386 | No |
| AGAP1 | 1.25 | 0.043686386 | No |
| PLCL2 | -1.75 | 0.044144496 | No |
| KLF16 | -0.53 | 0.044144496 | No |
| CXorf56 | 0.67 | 0.044144496 | No |
| CREG1 | -1.05 | 0.044144496 | No |
| STAG3L4 | 1.33 | 0.044144496 | Yes |
| FAM127A | 0.83 | 0.044380943 | No |
| ADSSL1 | -2.13 | 0.044446569 | No |
| PRKAB2 | -1.07 | 0.044446569 | No |
| VPS35 | 0.55 | 0.044469834 | No |
| BHLHB9 | 1.24 | 0.045042557 | No |
| PRUNE2 | 2.76 | 0.045042557 | No |
| PIAS3 | -0.75 | 0.045042557 | No |
| MYH9 | -0.83 | 0.045042557 | No |
| FUT9 | 2.74 | 0.045316351 | No |
| DUS4L | 0.98 | 0.045340118 | No |
| POLR3C | -0.65 | 0.045340118 | No |
| KLF8 | 1.62 | 0.045340118 | No |
| TOX | -2.17 | 0.045340118 | No |
| CHD1L | -1.09 | 0.045340118 | No |
| RSL24D1 | 0.45 | 0.045340118 | No |
| CTTN | -0.73 | 0.045340118 | No |
| TCTN2 | 0.47 | 0.045340118 | No |
| RTN1 | 1.73 | 0.045340118 | No |
| SCD5 | 1.20 | 0.045340118 | No |
| DRG2 | 0.62 | 0.045340118 | No |
| PPP1R12A | -0.73 | 0.045340118 | No |
| ARMCX2 | 1.55 | 0.045340118 | Yes |
| SHB | -1.27 | 0.045734033 | No |
| TBX19 | -0.63 | 0.046823047 | No |
| NTN1 | 2.26 | 0.046896246 | No |
| CHST10 | 1.03 | 0.046896246 | No |
| CYB5D2 | 1.17 | 0.046896246 | No |
| ULK2 | 0.89 | 0.047008242 | No |
| PARP14 | -0.62 | 0.047008242 | No |
| C11orf71 | 1.15 | 0.047029515 | No |
| AQP1 | 3.57 | 0.047029515 | No |
| TRAP1 | 0.57 | 0.047029515 | No |
| SFT2D2 | -0.68 | 0.047029515 | No |
| NOD2 | -1.15 | 0.047029515 | No |
| ORC3 | 0.54 | 0.047029515 | No |
| FAM76A | 0.90 | 0.047029515 | Yes |
| RRP15 | -0.49 | 0.047252533 | No |
| DUT | 0.67 | 0.047270057 | No |
| SAMD4A | 1.10 | 0.047270057 | No |
| PREX1 | -1.28 | 0.047270057 | Yes |
| SLC8B1 | -0.67 | 0.047272389 | Yes |
| GBAS | 0.74 | 0.047272389 | Yes |
| EZR | 1.05 | 0.047514177 | No |
| POLE | -0.75 | 0.047514177 | No |
| NCS1 | 0.49 | 0.047514177 | No |
| PITPNA-AS1 | 0.80 | 0.047514177 | No |
| FAM127B | 0.71 | 0.047514177 | No |
| HINT3 | 0.63 | 0.047514177 | No |
| SRSF8 | -0.64 | 0.047514177 | No |
| TPM2 | -1.44 | 0.047514177 | No |
| PPP6R1 | -0.46 | 0.047514177 | No |
| SEL1L3 | -2.61 | 0.047514177 | No |
| EFHD2 | -1.17 | 0.04844269 | No |
| HIC2 | -0.68 | 0.048475081 | No |
| FAM122C | 1.87 | 0.048475081 | No |
| DPH6 | 0.72 | 0.048475081 | No |
| GLOD4 | 0.65 | 0.048475081 | No |
| CLIC4 | -1.26 | 0.048475081 | No |
| SMIM3 | -1.38 | 0.048566505 | No |
| LOC257396 | 0.97 | 0.048816001 | No |
| PEX12 | 1.00 | 0.048816001 | No |
| ZNF23 | 1.16 | 0.048816001 | No |
| ANKRD27 | -1.01 | 0.048816001 | No |
| PPP1R3F | 0.58 | 0.048816001 | No |
| PAK1 | -1.12 | 0.048816001 | No |
| ADRA1A | 1.50 | 0.048816001 | No |
| TARSL2 | 0.93 | 0.048816001 | No |
| EML4 | -1.13 | 0.048816001 | No |
| HOXC10 | 3.20 | 0.048816001 | Yes |
| LOC284242 | 0.87 | 0.048998558 | No |
| ANGPTL4 | -0.88 | 0.048998558 | No |
| SUSD1 | -1.71 | 0.048998558 | No |
| COLEC11 | -1.54 | 0.048998558 | No |
| LOC100996419 | 1.07 | 0.048998558 | Yes |
| PCSK1N | 2.37 | 0.049293421 | No |
| HNRNPU-AS1 | -1.41 | 0.049293421 | No |
| VPS45 | -0.46 | 0.049538644 | No |
| IDH3A | 1.18 | 0.049580577 | No |
| C16orf91 | 0.52 | 0.049580577 | No |
| ARMC8 | -0.61 | 0.049580577 | No |
| LRRC8C | -0.78 | 0.049580577 | No |
| SLC2A1 | -0.91 | 0.049856921 | No |
| ATP5A1 | 0.51 | 0.049856921 | No |
| MTHFR | -0.83 | 0.049856921 | No |
| MORF4L2 | 0.54 | 0.049856921 | No |

**Supplementary Table 5**. **Enrichment of chromosomal regions with significantly down-regulated genes in spinal ependymoma (EPN).**

| **Chr** | **Start** | **End** | **FDR** |
| --- | --- | --- | --- |
| **PEDIATIRC SEPN** |  |  |  |
| 17 | 24924620 | 24965904 | 0.000938879 |
| 14 | 92049890 | 95629886 | 0.000938879 |
| 2 | 241174825 | 241408398 | 0.00289133 |
| 14 | 92049890 | 92651980 | 0.00289133 |
| 9 | 135326038 | 136472250 | 0.005137891 |
| 4 | 42105147 | 56996602 | 0.005137891 |
| 4 | 42105147 | 47123218 | 0.005137891 |
| 14 | 94148467 | 95629886 | 0.006815407 |
| 3 | 117011854 | 122621336 | 0.023385223 |
| 17 | 39828178 | 41461547 | 0.023542759 |
| 13 | 21143170 | 29067721 | 0.032623842 |
| 16 | 1299181 | 2186434 | 0.034487861 |
| 21 | 43346374 | 45786779 | 0.048064058 |
| 20 | 56397651 | 61432728 | 0.048553488 |
| **ADULT SEPN** |  |  |  |
| 1 | 144287346 | 179258670 | 1.94E-05 |
| 1 | 165776874 | 166550288 | 3.17E-05 |
| 1 | 144287346 | 145234067 | 5.20E-05 |
| 1 | 153571683 | 153975425 | 6.47E-05 |
| 1 | 166320621 | 166550288 | 6.47E-05 |
| 22 | 20101693 | 20637217 | 0.000223944 |
| 11 | 65096396 | 111142345 | 0.00041711 |
| 19 | 1803399 | 2179346 | 0.0004433 |
| 3 | 148588023 | 148617196 | 0.000489154 |
| 4 | 110700897 | 110844078 | 0.000489154 |
| 11 | 65096396 | 65174975 | 0.000879705 |
| 22 | 20381828 | 20637217 | 0.000879705 |
| 1 | 144287346 | 144322294 | 0.000879705 |
| 1 | 145093314 | 145234067 | 0.000879705 |
| 14 | 52311662 | 53493362 | 0.001306405 |
| 19 | 13076721 | 13478038 | 0.001869356 |
| 1 | 1233810 | 1368125 | 0.004234303 |
| 22 | 34267296 | 35113958 | 0.00494864 |
| 22 | 44277383 | 45512816 | 0.00494864 |
| 11 | 75203923 | 76862581 | 0.007150139 |
| 12 | 56503617 | 63375459 | 0.010841601 |
| 11 | 69922292 | 71627797 | 0.012892912 |
| 3 | 17026221 | 25799997 | 0.013855279 |
| 1 | 204710414 | 216577948 | 0.018828448 |
| 2 | 114364007 | 127581334 | 0.021215759 |
| 2 | 219779759 | 223626872 | 0.021215759 |
| 12 | 123374915 | 131923460 | 0.022333408 |
| 16 | 86421131 | 88333811 | 0.023567858 |
| 1 | 209566580 | 216577948 | 0.024812405 |
| 9 | 35671989 | 38059249 | 0.028993303 |
| 13 | 99432294 | 112800864 | 0.031571914 |
| 11 | 102438037 | 111142345 | 0.035936773 |
| 1 | 11768367 | 15629426 | 0.048789603 |

**Supplementary Table 6**. **Putative miRNA–mRNA interactions in pediatric spinal ependymoma (EPN).**

| **miRNA** | **Gene** | **R** | **FDR** | **Experiments** | **Support Type** | **References (PMID)** |
| --- | --- | --- | --- | --- | --- | --- |
| hsa-miR-10a-5p | IL1RAPL1 | -0.74 | 2.58E-05 |  |  |  |
| hsa-miR-10a-5p | RNF112 | -0.69 | 1.64E-04 |  |  |  |
| hsa-miR-10a-5p | HDAC4 | -0.68 | 2.01E-04 |  |  |  |
| hsa-miR-124-3p | ST5 | -0.66 | 4.18E-04 |  |  |  |
| hsa-miR-10a-5p | ELAVL3 | -0.66 | 4.43E-04 |  |  |  |
| hsa-miR-27b-3p | KIAA2022 | -0.65 | 6.28E-04 |  |  |  |
| hsa-miR-23b-3p | MESP1 | -0.64 | 9.75E-04 |  |  |  |
| hsa-miR-10b-5p | RNF112 | -0.62 | 1.58E-03 |  |  |  |
| hsa-miR-10a-5p | GABRB1 | -0.61 | 1.89E-03 |  |  |  |
| hsa-miR-23b-3p | KCNQ5 | -0.61 | 2.02E-03 |  |  |  |
| hsa-miR-27b-3p | KIF21B | -0.61 | 2.23E-03 |  |  |  |
| hsa-miR-10a-5p | BCL2L2 | -0.60 | 2.33E-03 |  |  |  |
| hsa-miR-10b-5p | IL1RAPL1 | -0.60 | 2.72E-03 |  |  |  |
| hsa-miR-10a-5p | TBX5 | -0.59 | 2.96E-03 |  |  |  |
| hsa-miR-10a-5p | ALPL | -0.59 | 3.27E-03 |  |  |  |
| hsa-miR-10a-5p | CAMK2B | -0.59 | 3.64E-03 |  |  |  |
| hsa-miR-153-3p | GAREM | -0.58 | 3.68E-03 |  |  |  |
| hsa-miR-10b-5p | MTSS1L | -0.58 | 4.18E-03 |  |  |  |
| hsa-miR-10b-5p | CAMK2B | -0.58 | 4.42E-03 |  |  |  |
| hsa-miR-124-3p | VANGL1 | -0.58 | 4.69E-03 | Microarray | Functional MTI (Weak) | 18668037 |
| hsa-miR-10a-5p | FXR2 | -0.57 | 4.95E-03 |  |  |  |
| hsa-miR-10b-5p | CADM2 | -0.57 | 5.09E-03 |  |  |  |
| hsa-miR-27b-3p | CNTNAP2 | -0.57 | 5.43E-03 |  |  |  |
| hsa-miR-10b-5p | GABRB1 | -0.57 | 5.78E-03 |  |  |  |
| hsa-miR-124-3p | TSPAN6 | -0.56 | 7.17E-03 | Microarray | Functional MTI (Weak) | 18668037 |
| hsa-miR-23b-3p | GPSM1 | -0.56 | 7.58E-03 |  |  |  |
| hsa-miR-23b-3p | SCN8A | -0.55 | 8.00E-03 |  |  |  |
| hsa-miR-23b-3p | SS18L1 | -0.55 | 8.30E-03 |  |  |  |
| hsa-miR-124-3p | ITPKB | -0.55 | 8.31E-03 |  |  |  |
| hsa-miR-23b-3p | MGAT3 | -0.55 | 8.60E-03 |  |  |  |
| hsa-miR-124-3p | AFF1 | -0.55 | 8.67E-03 |  |  |  |
| hsa-miR-23b-3p | MARCKSL1 | -0.55 | 8.67E-03 |  |  |  |
| hsa-miR-10a-5p | GRM3 | -0.55 | 8.87E-03 |  |  |  |
| hsa-miR-124-3p | STK35 | -0.55 | 9.05E-03 | PAR-CLIP | Functional MTI (Weak) | 26701625 |
| hsa-miR-10a-5p | ADAMTS2 | -0.55 | 9.07E-03 |  |  |  |
| hsa-miR-23b-3p | MTSS1 | -0.55 | 9.23E-03 |  |  |  |
| hsa-miR-153-3p | MATN2 | -0.54 | 9.55E-03 |  |  |  |
| hsa-miR-10a-5p | USB1 | -0.54 | 9.55E-03 |  |  |  |
| hsa-miR-27b-3p | MAPT | -0.54 | 9.76E-03 |  |  |  |
| hsa-miR-27b-3p | EEPD1 | -0.54 | 9.77E-03 |  |  |  |
| hsa-miR-26a-5p | TBC1D4 | -0.54 | 1.03E-02 |  |  |  |
| hsa-miR-124-3p | EFNB3 | -0.54 | 1.08E-02 |  |  |  |
| hsa-miR-124-3p | RBM24 | -0.53 | 1.17E-02 | Microarray | Functional MTI (Weak) | 18668037 |
| hsa-miR-10a-5p | ONECUT2 | -0.53 | 1.18E-02 |  |  |  |
| hsa-miR-124-3p | KIAA0895 | -0.53 | 1.18E-02 |  |  |  |
| hsa-miR-124-3p | TMED1 | -0.53 | 1.21E-02 | Microarray | Functional MTI (Weak) | 18668037 |
| hsa-miR-124-3p | GRSF1 | -0.53 | 1.27E-02 | Proteomics;Microarray | Functional MTI (Weak) | 18668037 |
| hsa-miR-10b-5p | HDAC4 | -0.53 | 1.29E-02 |  |  |  |
| hsa-miR-27b-3p | SNAP25 | -0.53 | 1.30E-02 |  |  |  |
| hsa-miR-23b-3p | KCNK3 | -0.52 | 1.43E-02 |  |  |  |
| hsa-miR-124-3p | PTTG1IP | -0.52 | 1.47E-02 | Microarray | Functional MTI (Weak) | 15685193; 18668037 |
| hsa-miR-10b-5p | HTT | -0.52 | 1.53E-02 |  |  |  |
| hsa-miR-10a-5p | TGOLN2 | -0.52 | 1.59E-02 |  |  |  |
| hsa-miR-27b-3p | PKIA | -0.51 | 1.61E-02 |  |  |  |
| hsa-miR-124-3p | NAV2 | -0.51 | 1.62E-02 |  |  |  |
| hsa-miR-10a-5p | PPP3CB | -0.51 | 1.63E-02 |  |  |  |
| hsa-miR-153-3p | SLC44A1 | -0.51 | 1.64E-02 |  |  |  |
| hsa-miR-10b-5p | PPP3CB | -0.51 | 1.65E-02 | CLASH | Functional MTI (Weak) | 23622248 |
| hsa-miR-10a-5p | VASH1 | -0.51 | 1.66E-02 |  |  |  |
| hsa-miR-10a-5p | SLC35G1 | -0.51 | 1.72E-02 |  |  |  |
| hsa-miR-27b-3p | TMTC4 | -0.51 | 1.79E-02 |  |  |  |
| hsa-miR-124-3p | ZFAND3 | -0.51 | 1.79E-02 |  |  |  |
| hsa-miR-10a-5p | RTN4R | -0.51 | 1.80E-02 |  |  |  |
| hsa-miR-23b-3p | IGDCC3 | -0.51 | 1.81E-02 |  |  |  |
| hsa-miR-124-3p | CUL5 | -0.51 | 1.81E-02 |  |  |  |
| hsa-miR-124-3p | SGMS2 | -0.51 | 1.81E-02 |  |  |  |
| hsa-miR-124-3p | AR | -0.51 | 1.88E-02 | Luciferase reporter assay//qRT-PCR//Western blot | Functional MTI | 22386953 |
| hsa-miR-124-3p | TRMT10A | -0.50 | 1.98E-02 |  |  |  |
| hsa-miR-10a-5p | HERC6 | -0.50 | 2.02E-02 |  |  |  |
| hsa-miR-23b-3p | PKIA | -0.50 | 2.03E-02 |  |  |  |
| hsa-miR-27b-3p | SBK1 | -0.50 | 2.05E-02 |  |  |  |
| hsa-miR-27b-3p | ACVR1C | -0.50 | 2.06E-02 |  |  |  |
| hsa-miR-153-3p | PCTP | -0.50 | 2.07E-02 |  |  |  |
| hsa-miR-153-3p | ACOT13 | -0.50 | 2.07E-02 |  |  |  |
| hsa-miR-23b-3p | SOX11 | -0.50 | 2.08E-02 |  |  |  |
| hsa-miR-124-3p | CTXN1 | -0.50 | 2.14E-02 | PAR-CLIP | Functional MTI (Weak) | 26701625 |
| hsa-miR-124-3p | FLNB | -0.50 | 2.15E-02 |  |  |  |
| hsa-miR-153-3p | CIB2 | -0.50 | 2.24E-02 |  |  |  |
| hsa-miR-10a-5p | PRRT3 | -0.50 | 2.25E-02 |  |  |  |
| hsa-miR-124-3p | C2orf68 | -0.50 | 2.25E-02 |  |  |  |
| hsa-miR-23b-3p | SAMD12 | -0.50 | 2.25E-02 |  |  |  |
| hsa-miR-153-3p | FAM168A | -0.50 | 2.25E-02 |  |  |  |
| hsa-miR-23b-3p | APPBP2 | -0.50 | 2.26E-02 |  |  |  |
| hsa-miR-23b-3p | PRPF40A | -0.49 | 2.33E-02 |  |  |  |
| hsa-miR-27b-3p | BEND4 | -0.49 | 2.38E-02 |  |  |  |
| hsa-miR-23b-3p | SAMD8 | -0.49 | 2.58E-02 |  |  |  |
| hsa-miR-144-5p | PSD2 | -0.49 | 2.64E-02 |  |  |  |
| hsa-miR-23b-3p | CBFA2T3 | -0.49 | 2.64E-02 |  |  |  |
| hsa-miR-10a-5p | BSN | -0.49 | 2.72E-02 |  |  |  |
| hsa-miR-153-3p | CITED2 | -0.48 | 2.86E-02 |  |  |  |
| hsa-miR-124-3p | KIAA1958 | -0.48 | 2.91E-02 |  |  |  |
| hsa-miR-23b-3p | C2orf69 | -0.48 | 2.93E-02 | PAR-CLIP | Functional MTI (Weak) | 21572407 |
| hsa-miR-153-3p | TNIK | -0.48 | 2.99E-02 |  |  |  |
| hsa-miR-23b-3p | DOK6 | -0.48 | 3.05E-02 |  |  |  |
| hsa-miR-124-3p | KIAA0556 | -0.48 | 3.14E-02 |  |  |  |
| hsa-miR-23b-3p | MTMR9 | -0.48 | 3.18E-02 |  |  |  |
| hsa-miR-27b-3p | CBFA2T3 | -0.48 | 3.20E-02 |  |  |  |
| hsa-miR-153-3p | FRMD5 | -0.48 | 3.23E-02 |  |  |  |
| hsa-miR-10a-5p | SERAC1 | -0.47 | 3.25E-02 |  |  |  |
| hsa-miR-153-3p | XKR4 | -0.47 | 3.26E-02 |  |  |  |
| hsa-miR-10a-5p | MTSS1L | -0.47 | 3.26E-02 |  |  |  |
| hsa-miR-124-3p | SLC5A3 | -0.47 | 3.29E-02 |  |  |  |
| hsa-miR-124-3p | MATN2 | -0.47 | 3.38E-02 |  |  |  |
| hsa-miR-10a-5p | NFAT5 | -0.47 | 3.47E-02 |  |  |  |
| hsa-miR-153-3p | TP53INP2 | -0.47 | 3.47E-02 |  |  |  |
| hsa-miR-124-3p | IL17RD | -0.47 | 3.49E-02 |  |  |  |
| hsa-miR-23b-3p | TNKS2 | -0.47 | 3.65E-02 |  |  |  |
| hsa-miR-10a-5p | SLC38A2 | -0.47 | 3.65E-02 |  |  |  |
| hsa-miR-124-3p | NR4A3 | -0.47 | 3.66E-02 | Microarray | Functional MTI (Weak) | 18668037 |
| hsa-miR-124-3p | PAX3 | -0.47 | 3.71E-02 |  |  |  |
| hsa-miR-124-3p | DSG2 | -0.47 | 3.72E-02 | Proteomics;Microarray | Functional MTI (Weak) | 18668037 |
| hsa-miR-10a-5p | NCOR2 | -0.47 | 3.72E-02 | qRT-PCR//Western blot | Functional MTI | 21212796 |
| hsa-miR-124-3p | FAM117A | -0.47 | 3.72E-02 |  |  |  |
| hsa-miR-27b-3p | APPBP2 | -0.47 | 3.73E-02 | PAR-CLIP | Functional MTI (Weak) | 23592263; 24398324; 22012620; 26701625 |
| hsa-miR-23b-3p | TLK1 | -0.47 | 3.76E-02 |  |  |  |
| hsa-miR-10a-5p | CADM2 | -0.47 | 3.79E-02 |  |  |  |
| hsa-miR-10a-5p | LRRC8B | -0.46 | 3.83E-02 |  |  |  |
| hsa-miR-27b-3p | NAV1 | -0.46 | 3.83E-02 |  |  |  |
| hsa-miR-10b-5p | GRM3 | -0.46 | 3.98E-02 |  |  |  |
| hsa-miR-23b-3p | MBOAT7 | -0.46 | 4.02E-02 |  |  |  |
| hsa-miR-10b-5p | NFIX | -0.46 | 4.13E-02 |  |  |  |
| hsa-miR-23b-3p | PJA1 | -0.46 | 4.13E-02 |  |  |  |
| hsa-miR-23b-3p | KDM4A | -0.46 | 4.16E-02 |  |  |  |
| hsa-miR-27b-3p | CHST1 | -0.46 | 4.16E-02 |  |  |  |
| hsa-miR-124-3p | COTL1 | -0.46 | 4.16E-02 |  |  |  |
| hsa-miR-124-3p | EFHC1 | -0.46 | 4.18E-02 |  |  |  |
| hsa-miR-124-3p | KIAA2013 | -0.46 | 4.18E-02 |  |  |  |
| hsa-miR-153-3p | ITPR1 | -0.46 | 4.24E-02 |  |  |  |
| hsa-miR-124-3p | RBM20 | -0.46 | 4.25E-02 |  |  |  |
| hsa-miR-23b-3p | EPHB2 | -0.46 | 4.33E-02 |  |  |  |
| hsa-miR-27b-3p | GRIN2D | -0.46 | 4.35E-02 |  |  |  |
| hsa-miR-124-3p | ANTXR2 | -0.46 | 4.36E-02 |  |  |  |
| hsa-miR-124-3p | SYNPO2 | -0.46 | 4.40E-02 |  |  |  |
| hsa-miR-124-3p | DTNA | -0.45 | 4.45E-02 |  |  |  |
| hsa-miR-27b-3p | DIRAS1 | -0.45 | 4.45E-02 |  |  |  |
| hsa-miR-27b-3p | SEMA7A | -0.45 | 4.46E-02 |  |  |  |
| hsa-miR-153-3p | TGFBR2 | -0.45 | 4.47E-02 |  |  |  |
| hsa-miR-27b-3p | FNDC4 | -0.45 | 4.48E-02 |  |  |  |
| hsa-miR-124-3p | DPH3 | -0.45 | 4.50E-02 |  |  |  |
| hsa-miR-27b-3p | FASN | -0.45 | 4.51E-02 | CLASH | Functional MTI (Weak) | 23622248 |
| hsa-miR-27b-3p | MTMR4 | -0.45 | 4.53E-02 |  |  |  |
| hsa-miR-27b-3p | KHSRP | -0.45 | 4.56E-02 | Western blot, luciferase assay | Functional MTI | 22615562 |
| hsa-miR-23b-3p | GXYLT1 | -0.45 | 4.56E-02 |  |  |  |
| hsa-miR-23b-3p | ACVR1C | -0.45 | 4.57E-02 |  |  |  |
| hsa-miR-124-3p | RALGPS2 | -0.45 | 4.67E-02 |  |  |  |
| hsa-miR-23b-3p | GPR155 | -0.45 | 4.67E-02 |  |  |  |
| hsa-miR-23b-3p | FAM117B | -0.45 | 4.68E-02 |  |  |  |
| hsa-miR-144-5p | SLC35A1 | -0.45 | 4.69E-02 |  |  |  |
| hsa-miR-23b-3p | UNC13A | -0.45 | 4.69E-02 |  |  |  |
| hsa-miR-23b-3p | LSAMP | -0.45 | 4.75E-02 |  |  |  |
| hsa-miR-27b-3p | BRSK1 | -0.45 | 4.76E-02 |  |  |  |
| hsa-miR-124-3p | SUMF1 | -0.45 | 4.78E-02 |  |  |  |
| hsa-miR-124-3p | ZBED3 | -0.45 | 4.78E-02 | Microarray | Functional MTI (Weak) | 15685193; 18668037 |
| hsa-miR-23b-3p | PKDCC | -0.45 | 4.80E-02 |  |  |  |
| hsa-miR-27b-3p | MAGI3 | -0.45 | 4.81E-02 |  |  |  |
| hsa-miR-124-3p | DIAPH1 | -0.45 | 4.82E-02 |  |  |  |
| hsa-miR-27b-3p | NRXN1 | -0.45 | 4.89E-02 |  |  |  |
| hsa-miR-10b-5p | TMEM132B | -0.45 | 4.90E-02 |  |  |  |
| hsa-miR-27b-3p | PHLPP2 | -0.45 | 4.94E-02 | HITS-CLIP | Functional MTI (Weak) | 22473208 |
